# Hydroxyl‐Rich Hyperbranched Polyglycerol Additive for Low‐Temperature Aqueous Zinc Batteries: Sustained and Efficient Dehydration and High‐Conductivity

**DOI:** 10.1002/advs.202516639

**Published:** 2025-11-19

**Authors:** Xiaoping Li, Tan Jin, Zhiqiao Wang, Yaoxuan Chen, Chunlong Yun, Jie Shang, Yan Ge, Tingli Lu, Wei Zhuang, Yue Ma, Zhenhui Qi

**Affiliations:** ^1^ School of Life Sciences Northwestern Polytechnical University Xi'an Shaanxi 710072 China; ^2^ State Key Laboratory of Structural Chemistry, Fujian Institute of Research on the Structure of Matter, Chinese Academy of Sciences Fuzhou Fujian 350002 China; ^3^ State Key Laboratory of Solidification Processing Center for Nano Energy Materials Center of Advanced Lubrication and Seal Materials School of Materials Science and Engineering Northwestern Polytechnical University and Shaanxi Joint Laboratory of Graphene (NPU) Xi'an Shaanxi 710072 China; ^4^ Southeast University Nanjing Jiangsu 210096 China; ^5^ State Key Laboratory of Digital Medical Engineering School of Biological Science and Medical Engineering Southeast University Nanjing Jiangsu 210096 China; ^6^ Shenzhen Research Institute of Northwestern Polytechnical University Shenzhen Guangdong 518057 China

**Keywords:** biomimicry, energy storage, ice growth, ion channels, polar environments

## Abstract

The development of aqueous Zn‐ion batteries operable at subzero temperatures is impeded by a number of design problems, including slow ion transport and interfacial instability. Drawing inspiration from marine fish adapted to polar waters, a bioinspired supramolecular additive hyperbranched polyglycerol, CDhPG is developed, that integrates dual biomimetic functions, thus overcoming the design limitations of Zn‐ion batteries. Specifically, CDhPG mimics the ice‐binding behavior of antifreeze proteins and the dehydration microenvironment of potassium ion channels, enabling simultaneous inhibition of ice growth and acceleration of Zn^2+^ desolvation. The hydroxyl‐rich architecture facilitates strong hydrogen bonding with ice surfaces, while its internal cavities promote selective Zn^2+^ coordination, thereby remodelling both the ice‐water and Zn‐electrolyte interfaces. As a result, the ZnCl_2_‐CDhPG electrolyte exhibits a suppressed freezing point (below −40 °C) and enables dendrite‐free Zn deposition. The Zn//Zn cells are found to deliver stable cycling over 900 hrs at −40 °C (5 mA cm^−2^, 43% DOD), and the full cells retain high capacity after 500 cycles at −40 °C. The bio‐inspired, dual‐functional strategy described here offers a generalizable approach for designing low‐temperature electrolytes in aqueous energy storage systems.

## Graphical abstract

Inspired by antifreeze proteins and ion channels in polar fish, β‐cyclodextrin–grafted hyperbranched polyglycerol (CDhPG) mimics interfacial water structuring and ion dehydration. The ice‐matching hydroxyl spacing (2.76 Å) enables IRI activity, while dendritic cavities tailor Zn^2+^ solvation and enhance low‐temperature conductivity for sustainable aqueous Zn batteries.

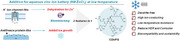

## 1 Introduction

Aqueous zinc‐ion batteries (ZIBs) present a compelling prospect for the low‐cost, upscaling of electrochemical energy storage, owing to the high volumetric capacity (5855 mAh∙cm^−3^) and cost‐effectiveness (≈1.35 USD$/lb) of Zn foil anode, as well as the inherent safety and ionic conductivity (1 S∙cm^−1^) of the aqueous electrolytes.^[^ ^1^, ^2^, ^3^, ^4^, ^5^, ^6^, ^7^, ^8^, ^9^, ^10^, ^11^, ^12^, ^13^ ^]^ However, their widespread application in low‐temperature environments—such as high‐latitude regions, polar areas, the deep sea, and outer space—remains challenging.^[^ ^14^, ^15^, ^16^, ^17^ ^]^ Aqueous ZIBs operating at low temperatures face several critical limitations: i) electrolyte freezing and ice crystal growth: the formation of ice nuclei followed by subsequent crystal growth disrupts ion transport pathways and induces structural damage within the electrolyte network,^[^ ^18^, ^19^, ^20^, ^21^, ^22^, ^23^, ^24^ ^]^ ii) drastically reduced ion mobility: reduced thermal energy slows Zn^2+^ desolvation, particularly with highly concentrated electrolytes, which leads to deteriorated rate capability and suppressed ionic diffusion, while the increased viscosity of the electrolyte further hinders ionic conductivity;^[^ ^25^, ^26^ ^]^ and, lastly iii) electrode/electrolyte interfacial failure: ice formation and heightened interfacial polarization accelerate dendrite growth, trigger parasitic side reactions, and ultimately compromise interfacial integrity.^[^ ^27^, ^28^, ^29^, ^30^ ^]^

To promote the development of aqueous ZIBs, tremendous efforts have been devoted to mitigating their low‐temperature limitations.^[^ ^31^, ^32^, ^33^, ^34^, ^35^, ^36^, ^37^, ^38^, ^39^, ^40^, ^41^, ^42^, ^43^, ^44^, ^45^, ^46^ ^]^ Organic solvent co‐electrolytes can effectively depress the freezing point of aqueous electrolytes (e.g., lowering it from 0 °C to below −60 °C with ethylene glycol or dimethyl sulfoxide) and thus maintain fluidity under extreme cold.^[^ ^47^, ^48^, ^49^, ^50^, ^51^, ^52^ ^]^ However, these solvents inherently introduce safety concerns due to flammability, volatility, and potential toxicity, as well as environmental persistence.^[^ ^53^, ^54^ ^]^ Salt concentration regulation, such as in “water‐in‐salt” electrolytes (e.g., 21 m Zn(TFSI)_2_ or ZnCl_2_), can also suppress water freezing by disrupting the hydrogen‐bond network, achieving freezing points below −20 °C.^[^ ^55^, ^56^ ^]^ Nevertheless, the resulting excessive viscosity and reduced ionic mobility stem from the high degree of ion‐ion association and solvent structure distortion, which impede Zn^2+^ diffusion.^[^ ^57^, ^58^, ^59^ ^]^ Moreover, such highly concentrated halide or bulky anion systems often cause severe corrosion of current collectors and cathode dissolution, while significantly increasing electrolyte cost.^[^ ^60^ ^]^ Molecular additives have recently emerged as a promising strategy, offering hydrogen‐bond modulation, solvation‐structure tuning, and in some cases anti‐freezing effects.^[^ ^61^ ^]^ Yet, most reported additives lack multifunctionality‐being effective at either ice suppression or dendrite inhibition but not both‐and exhibit limited long‐term compatibility with Zn anodes due to side reactions, decomposition, or instability of the additive‐derived interphase under repeated cycling. Accordingly, designing stable and efficient low‐temperature electrolytes without reducing water content is a key priority for aqueous zinc‐ion batteries and other electrochemical energy storage or wearable systems exposed to subzero conditions.^[^ ^43^, ^62^, ^63^ ^]^

To address these design challenges, we looked for inspiration from living organisms. Polar‐adapted fish have evolved remarkable adaptations to survive in subzero environments.^[^ ^64^ ^]^ In this study we demonstrate how key aspects of cold‐adapted physiology can be transferred to the design of low‐temperature aqueous battery systems. Polar‐adapted fish maintain high intracellular concentrations of salts, often exceeding 400 mm Na^+^ and 150 mm K^+^, which allows them to sustain osmotic balance and ionic conductivity in freezing conditions.^[^ ^65^, ^66^, ^67^, ^68^ ^]^ Efficient ion transport and exceptional antifreeze protection is achieved through specialized biological macromolecules. Ion channel proteins, such as potassium channels, involve precise dehydration mechanisms at the channel entrance—often involving conserved residues like threonine, glycine, and valine—that selectively strip hydration shells and facilitate rapid transmembrane ion conduction.^[^ ^69^, ^70^, ^71^, ^72^ ^]^ Concurrently, type III antifreeze proteins (AFPs) effectively inhibit ice crystal growth by adsorbing onto ice nucleation surfaces and disrupting the ice–water interface via hydrogen bonding and hydrophobic interactions. Accordingly, biomimetic strategies have emerged to enhance electrolyte performance at low temperatures.^[^ ^73^, ^74^, ^75^, ^76^, ^77^, ^78^ ^]^ For example, Sun et al. reported a synthetic ion channel‐inspired architecture that promotes Zn^2+^ desolvation and transport in aqueous ZIBs,^[^ ^72^ ^]^ and He et al. demonstrated that incorporating AFP‐mimicking structures could suppress ice formation and improve low‐temperature battery performancep.^[^ ^78^ ^]^ However, efforts to simultaneously harness both ion channel‐like dehydration and antifreeze protein‐like ice inhibition within a unified electrolyte system remain rare. Such a dual‐function strategy may hold the key to overcoming the long‐standing trade‐off between ion mobility and antifreeze protection in subzero electrochemical environments.^[^ ^79^ ^]^

**Figure 1:**
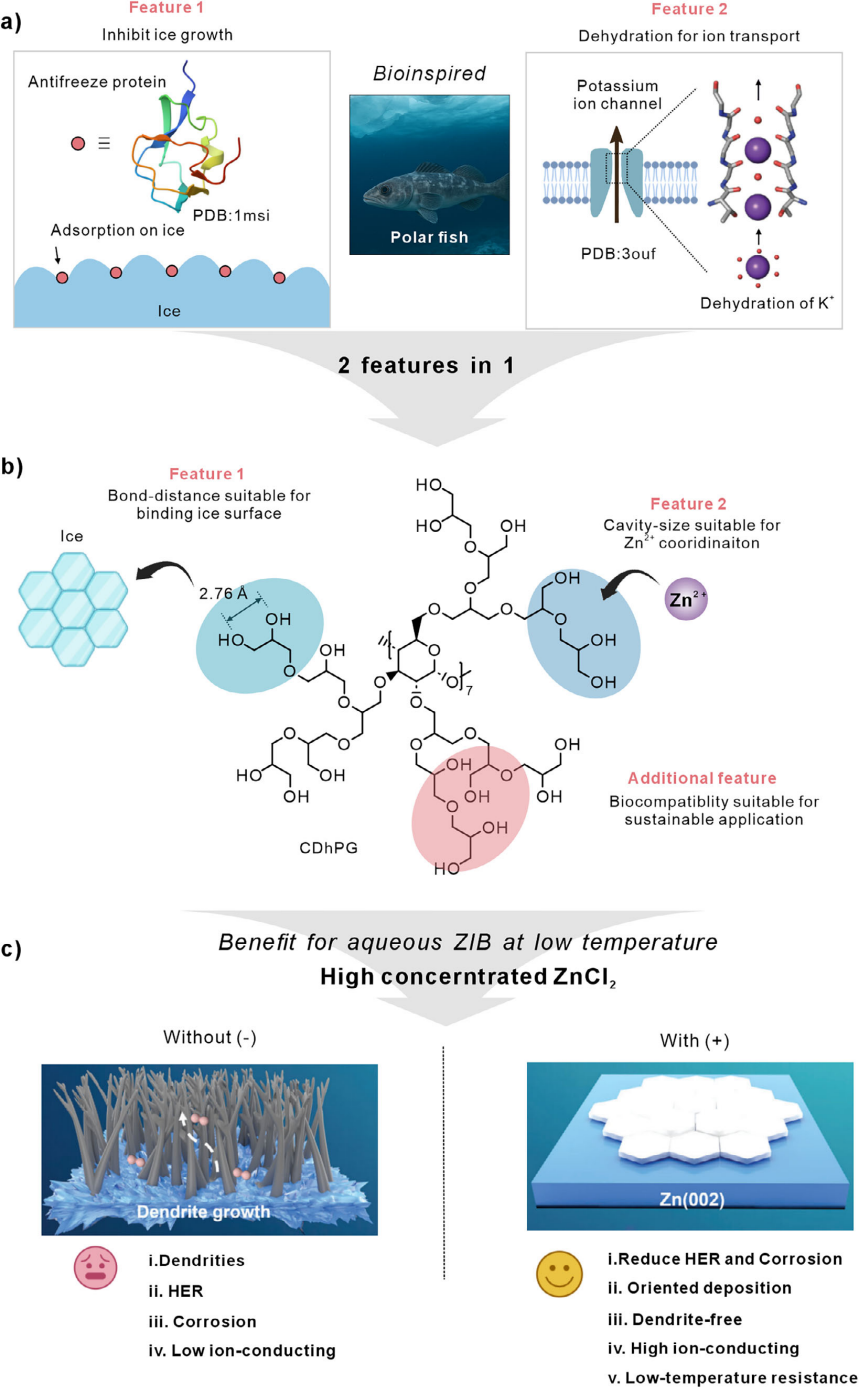
Bioinspired dual‐function design of β‐cyclodextrin–grafted hyperbranched polyglycerol (CDhPG) in low‐temperature aqueous Zn‐ion batteries. a): Electrolyte processes in polar‐adapted fish: (Feature 1) antifreeze proteins (e.g., PDB: 1msi) that inhibit ice growth by adsorbing to ice crystal surfaces, and (Feature 2) potassium ion channels (e.g., PDB: 3ouf) that facilitate K^+^ transport via selective dehydration mechanisms. b) CDhPG integrates both features into a single supramolecular design. The hydroxyl‐rich branches provide bond distances (≈2.76 Å) suitable for ice binding (Feature 1), and its internal cavities enable size‐matched coordination with Zn^2+^ (Feature 2). The molecule also exhibits good biocompatibility, supporting sustainable application. c): In highly concentrated ZnCl_2_ electrolyte, the incorporation of CDhPG improves performance at subzero temperatures by reducing hydrogen evolution and corrosion, guiding oriented Zn deposition, suppressing dendrite formation, and enhancing ionic conductivity—enabling stable Zn(002) plane growth. In contrast, the absence of CDhPG leads to dendritic Zn growth, severe HER, and poor ionic transport.

In this study, we assessed the potential of β‐cyclodextrin–grafted hyperbranched polyglycerol (CDhPG, **Figure** **1**) as a multifunctional electrolyte additive that transforms the low‐temperature performance landscape of aqueous zinc batteries. Its dendritic architecture, densely decorated with hydroxyl groups, forges strong, multivalent hydrogen‐bonding interactions with water. Remarkably, the O–O spacing between adjacent hydroxyl groups in polyglycerol (≈2.76 Å) is an almost perfect match to that of the hexagonal ice lattice (≈2.75 Å),^[^ ^77^ ^]^ enabling geometric locking that arrests ice nucleation and recrystallization with molecular precision. Beyond ice control, the globular, 3D topology—fundamentally distinct from linear hydroxylated polymers—creates a high‐density field of Zn^2^⁺ coordination sites, reshaping the solvation shell, disrupting extended hydrogen‐bond networks, and mitigating viscosity build‐up under subzero conditions. At the electrode–electrolyte interface, hydroxyl‐rich moieties exhibit an intrinsic affinity for Zn surfaces, spontaneously forming a protective interphase that suppresses dendrites and parasitic reactions. With a hydroxyl density exceeding 1.8 mmol ‐OH∙g^−1^, vastly surpassing linear analogues, CDhPG achieves robust interfacial anchoring and long‐term stability.^[^ ^80^, ^81^ ^]^ These structural and chemical synergies confer a dual benefit: enhanced ionic conductivity via hydrogen‐bond‐mediated water structuring and accelerated Zn^2^⁺ desolvation, together with preserved interfacial integrity throughout extended cycling. Crucially, CDhPG embodies sustainability without compromise: derived from a biocompatible polyglycerol framework, it is inherently green, non‐toxic, and chemically resilient, offering a viable alternative to organic or fluorinated low‐temperature additives. This rare confluence of ice recrystallization inhibition, solvation modulation, conductivity enhancement, and environmental compatibility defines CDhPG as a new material archetype for sustaining high‐performance aqueous ZIBs in the harshest cold environments.

## 2 Results and Discussion

### 2.1 CDhPG Inhibits Ice Crystal Growth and Lowers Freezing Point Synergistically

**Figure 2:**
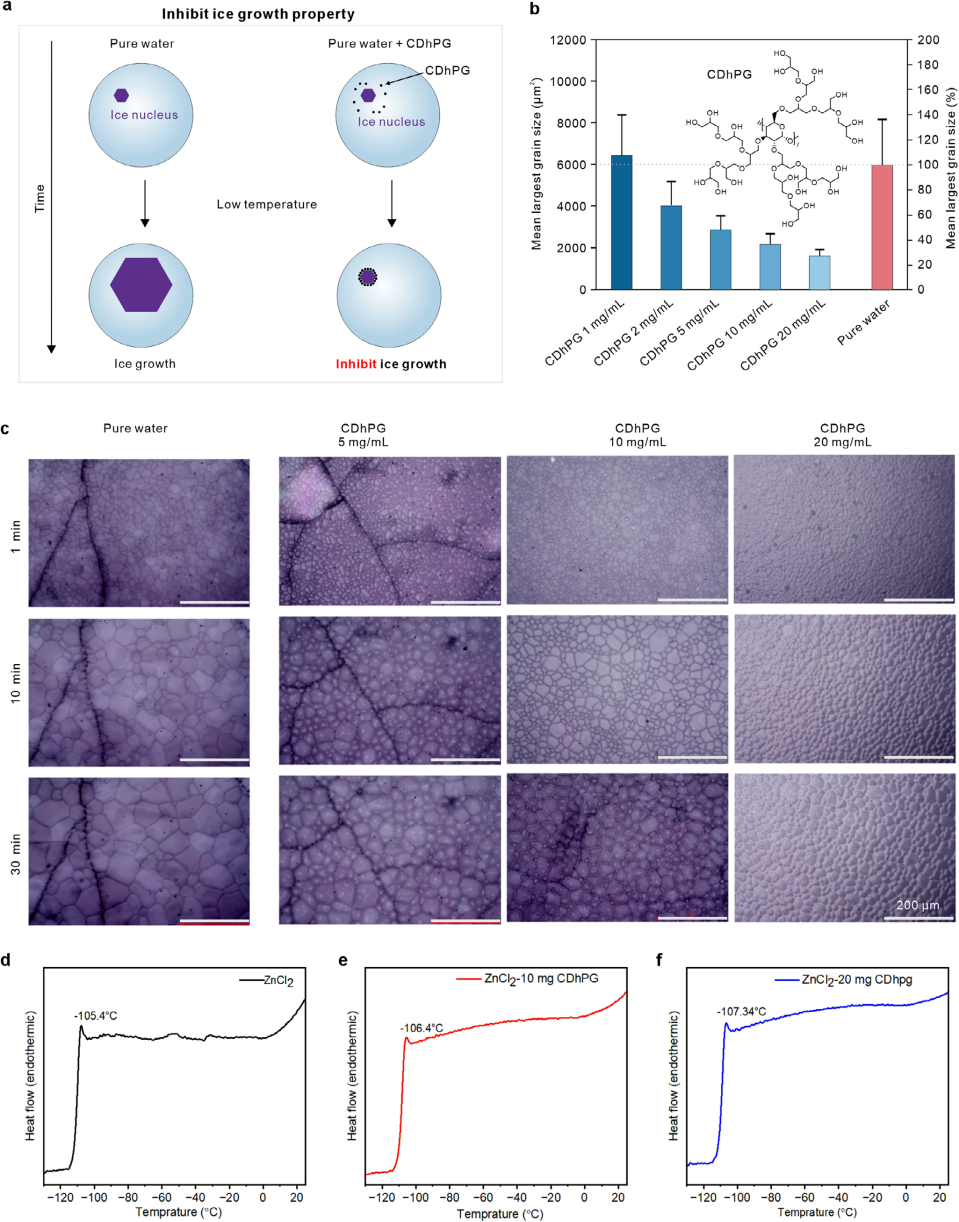
Ice recrystallization inhibition (IRI) performance of CDhPG. a) Schematic illustration of ice crystal growth in pure water and suppression of ice growth in the presence of CDhPG. b) Mean largest grain size (MLGS) of ice crystals in pure water and with varying CDhPG concentrations after annealing at −6 °C for 30 min; inset shows the molecular structure of CDhPG. c) Polarized optical microscopy images of ice crystals in pure water and in aqueous CDhPG solutions (5–20 mg∙mL^−1^) after annealing for 1, 10, and 30 min at −6 °C; scale bars: 200 µm. d–f) Differential scanning calorimetry (DSC) curves of 8 m ZnCl_2_ electrolyte without and with CDhPG (10 and 20 mg∙mL^−1^), showing freezing‐point depression and altered ice‐crystallization behavior.

Like the behavior of many reported AFPs,^[^ ^73^, ^74^ ^]^ the preferential adsorption of CDhPG onto the ice crystal surface imparts ice recrystallization inhibition (IRI) capability, which prevents the growth of larger ice crystals at the expense of smaller ones, thereby reducing the overall grain size (**Figure** **2**a). To assess the antifreeze functionality of CDhPG, we first examined its IRI activity in aqueous solution, a standard assay for evaluating antifreeze protein mimics. The utilized CDhPG was synthesized and thoroughly characterized to confirm its hyperbranched structure (see Material and Figure S1a,b, Supporting Information). In pure water, ice nuclei formed at subzero temperatures rapidly coarsened into large polyhedral crystals; in contrast, the presence of CDhPG markedly suppressed crystal growth. The mean largest grain size (MLGS) at 10 mg∙mL^−1^ CDhPG was 2100 ± 300 µm^2^, demonstrating its ability to inhibit ice recrystallization. Increasing the concentration to 20 mg∙mL^−1^ further reduced the MLGS to 1600 ± 300 µm^2^, indicating enhanced IRI performance. Quantitative analysis (Figure 2b) revealed a sharp decrease in MLGS with increasing CDhPG concentration, with reductions exceeding 70% at 10–20 mg∙mL^−1^ compared to pure water. Polarized optical microscopy (Figure 2c) further confirmed that CDhPG effectively suppressed ice coarsening over extended holding times (1–30 min), maintaining a fine‐grained, compact ice morphology even under prolonged subzero exposure. According to the anchoring model, this pronounced IRI activity originates from the dense hydroxyl arrays on the hyperbranched polyglycerol scaffold, which form multivalent hydrogen bonds with the quasi‐liquid layer on the ice surface, thereby blocking the incorporation of water molecules into the ice lattice. Notably, the calculated O‐O distance between adjacent hydroxyl groups in polyglycerol (≈2.76 Å) closely matches that of the hexagonal ice lattice (≈2.75 Å) (Figure 1), ensuring geometric complementarity akin to natural antifreeze proteins. As reported by He et al., the inhibition of ice growth by additives can create more accessible pathways for ion transport in ZIBs under low‐temperature conditions.^[^ ^78^ ^]^ Encouraged by its pronounced IRI activity, we found that incorporating CDhPG into highly concentrated ZnCl_2_ electrolytes further enhances their antifreeze capability. Specifically, adding 20 mg∙mL^−1^ CDhPG (less than 1 wt.%) to an 8 M ZnCl_2_ solution improved the already excellent low‐temperature tolerance of the electrolyte (Figure S2, Supporting Information). Differential scanning calorimetry (DSC) measurements (Figure 2d–f) revealed that this incorporation lowered the freezing point from −105.4 to −107.34 °C, underscoring the ability of the additive to extend liquid‐phase stability into deeper subzero regimes through synergistic IRI activity and hydrogen‐bond‐mediated suppression of ice nucleation.

The inhibitory effect of CDhPGs on ice growth was further examined by tracking the proportion of ice over time. We carried out non‐equilibrium NVT simulations to observe the time‐dependent evolution of CDhPG at the water/ice interface. As shown in Figure S3 (Supporting Information), at 267K, the CDhPG can actively restricts the crystallization of liquid water into the ice phase by inserting the side chain hyperbranched polyglycerol into the ice surface. Meanwhile, the inhibitory effect of CDhPG ice growth was also examined by tracking the evolution of hydrogen bond density over time. Without CDhPG, the hydrogen bond density appears a pronounced uptrend due to robust ice formation in Figure S3b (Supporting Information). With CDhPG, its growth significantly weakened. This comparison underscores the role of CDhPG in limiting ice growth through suppressing the hydrogen‐bonded network.

### 2.2 Dehydration‐Mediated Ion Transport Enhancement

**Figure 3:**
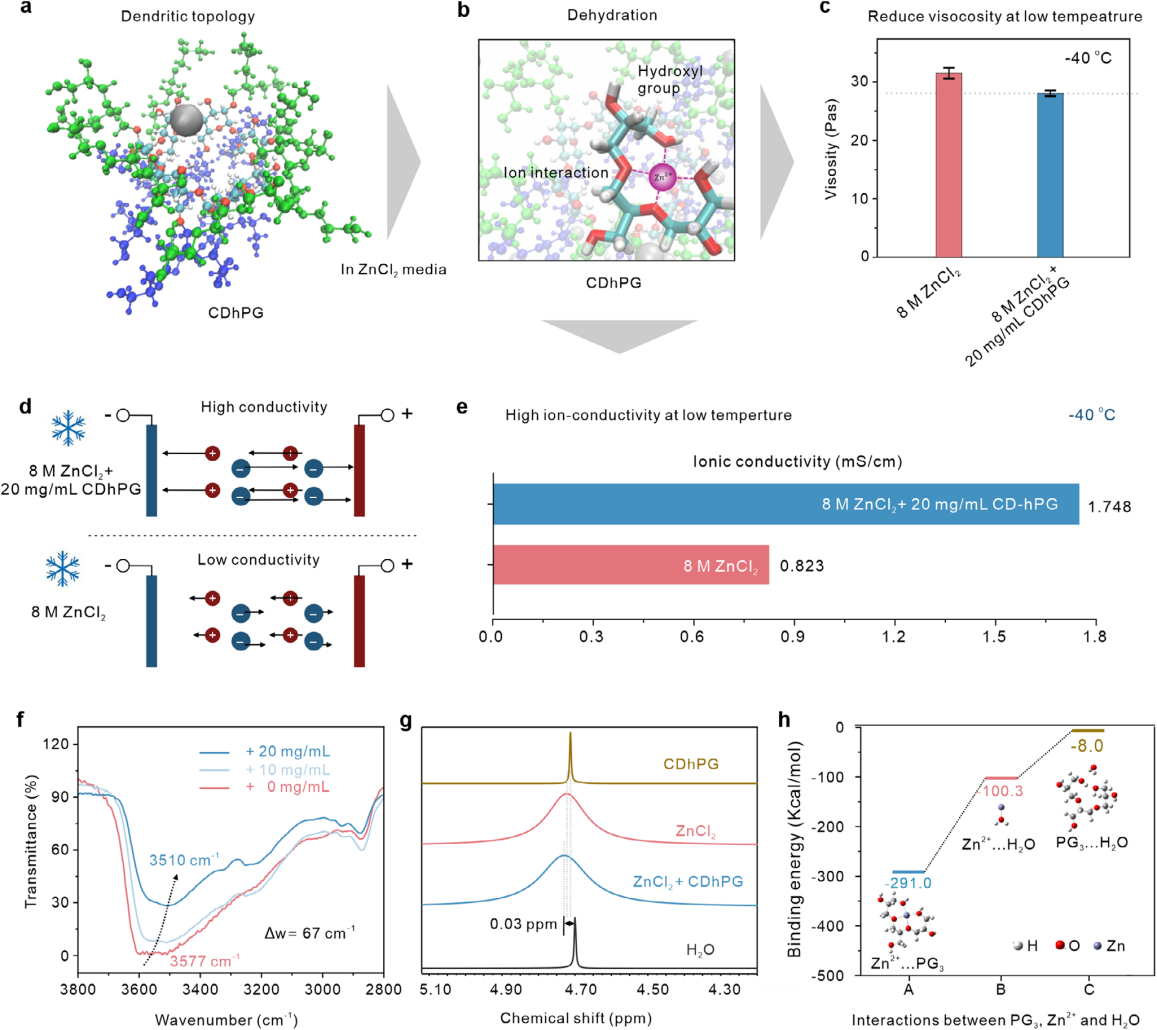
Dehydration‐driven enhancement of low‐temperature ZnCl_2_ electrolyte performance by CDhPG. a) Dendritic topology of CDhPG providing abundant hydroxyl groups for ion coordination. b) Molecular dynamics snapshot showing strong Zn^2+^‐hydroxyl interactions that competitively dehydrate the solvation shell. c) Reduced viscosity at −40 °C upon addition of 20 mg∙mL^−1^ CDhPG to 8 m ZnCl_2_, enabling faster ion transport. d) Schematic of improved ionic mobility via dehydration‐mediated viscosity reduction. e) Ionic conductivity at −40 °C doubled compared to pristine electrolyte. f) FTIR O‐H stretching blue shift confirming weakened hydrogen‐bond network and Zn^2+^ desolvation. g) ^2^H NMR revealing altered water dynamics in the hydration shell. h) DFT‐calculated binding energies showing Zn^2+^‐PG_3_ (a trimer form of polyglycerol) interactions far exceeding Zn^2+^‐H_2_O, validating the dehydration mechanism.

Molecular simulations (**Figure** **3**a; Figures S4 and S5, Supporting Information) reveal that CDhPG adopts a dendritic, highly branched architecture densely decorated with hydroxyl groups. Therefore, when the fully hydrated Zn^2+^ (Zn^2+^ tend to coordinate with two or six H_2_O molecules to form the primary [Zn(H_2_O)_6_]^2+^/[Zn(H_2_O)_2_Cl_4_]^2−^) solvation shells) approaching the periphery of CDhPG, these ‐OH moieties on CDHPG engage Zn^2+^ ions through geometry favourable four hydrogen bonding and coordination (Figure 3b). As shown in the 3D image in Figure S6 (Supporting Information), the CDhPG molecule can alter H_2_O molecules from the Zn^2+^ primary solvation shell and competitively displace hydration waters and thin the Zn^2+^ solvation shell, thereby reducing the effective hydrodynamic radius and facilitating ion transport under subzero conditions. Specifically, the electrostatic potential difference of the glycerol moiety and cyclodextrin core (Figure S4, Supporting Information) are particularly beneficial for the reduction of the electrostatic Zn^2+^ and accelerate the rapid transfer of Zn^2+^.

Consequently, we noted in the viscosity measurements (Figure 3c) that at −40 °C, the viscosity of pristine 8 m ZnCl_2_ (≈30 Pa∙s^−1^) decreases to ≈23 Pa∙s^−1^ upon the addition of 20 mg∙mL^−1^ CDhPG, indicating partial disruption of the ion‐water network and improved fluidity. In support of this, the transport schematic (Figure 3d) and conductivity data (Figure 3e) show that dehydration and suppression of water freezing result in a more than twofold increase in conductivity at −40 °C, from 0.823 to 1.748 mS∙cm^−1^. Spectroscopic evidence further corroborates the dehydration mechanism. FTIR spectra exhibit a blue shift of the O‐H stretching band from 3577 to 3510 cm^−1^ (Δν ≈ 67 cm^−1^) upon CDhPG incorporation (Figure 3f; Figure S7, Supporting Information), indicating a weakened hydrogen‐bond network and altered water coordination. Likewise, ^1^H NMR shows the ^2^H peak of Zn^2+^ has a 0.03 ppm shift (Figure 3g), consistent with the removal of water from the first solvation shell. Density functional theory calculations (Figure 3h) further reveal a strong thermodynamic preference for hydroxyl coordination: E_bind_ (Zn^2+^‐PG_3_) reached −291.0 kcal∙mol^−1^, which far exceeds that of Zn and water (E_bind_ (Zn^2+^‐H_2_O) = −100.3 kcal∙mol^−1^); in contrast, the degree of water binding of the polyglycerol moiety is comparatively weak (E_bind_(PG_3_‐H_2_O) = −8.0 kcal∙mol^−1^), underscoring the affinity of Zn^2+^ for multidentate hydroxyl ligands over water molecules. To clarify the ion transport mechanism, we performed NPT molecular dynamics simulations at different salt concentrations and calculated the mean square displacement (MSD) of both Zn^2+^ ions and H_2_O molecules (Figure S8, Supporting Information). The MSD results clearly demonstrate that the presence of CDhPG markedly enhances the mobility of both species. The MD simulations of Zn^2+^ adsorption behavior on CDhPG and β‐CD in ZnCl_2_ aqueous solution further rule out the possibility of Zn^2+^ directly passing through the cyclodextrin cavity of CDhPG (see Figures S9 and S10; Videos S1 and S2, Supporting Information). It is worthy noting that in the highly concentrated 8 m ZnCl_2_ electrolyte, Zn^2+^ ions predominantly exist as chloro‐aqua coordination complexes such as [ZnCl_4_]^2−^, [ZnCl_3_(H_2_O)]^−^, and [ZnCl_2_(H_2_O)_2_], rather than as fully hydrated [Zn(H_2_O)_6_]^2+^ species. Consequently, interfacial ion transport is more accurately described by a ligand‐exchange mechanism. The CDhPG molecules participate in dynamic coordination exchange with both H_2_O and Cl^−^ ligands in the primary solvation shell of Zn^2+^. The abundant hydroxyl and ether oxygen sites of CDhPG can transiently coordinate with Zn^2+^, partially replacing water or chloride ligands to form labile Zn–O(CDhPG) interactions. This competitive coordination weakens the Zn‐Cl and Zn‐O(H_2_O) bonds, thereby modulating the local solvation environment and lowering the desolvation energy barrier at the electrode interface. Such ligand exchange not only facilitates Zn^2+^ desolvation and migration but also suppresses the formation of rigid solvation cages, ultimately enabling enhanced faster charge transfer under subzero conditions.

### 2.3 Low‐Temperature Interfacial Engineering for Zn Anode Protection

Density functional theory (DFT) calculations was conducted to evaluate the adsorption mechanism of hydroxyl‐rich hyperbranched polyglycerol on Zn anode surface. The charge density difference analysis result in Figures S11–S13 (Supporting Information) show that the PG_3_ molecule (a trimer form of polyglycerol) has a higher charge transfer trend to Zn surface than that of water molecule, thereby manifest the stronger interaction between the PG_3_ molecule and Zn slab. Figure S11 (Supporting Information) shows that the PG_3_ molecule exhibits the higher absorption energy of −0.947 eV than water (−0.280 eV), indicating a stronger tendency of the former toward being adsorbed on the Zn surface.

**Figure 4:**
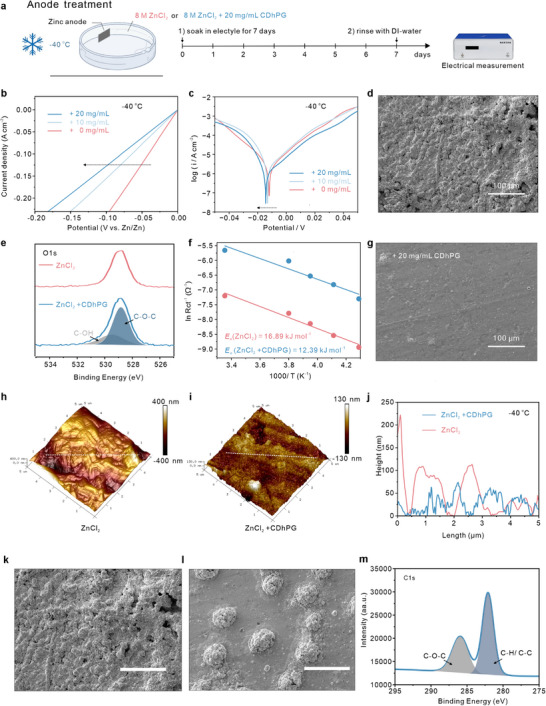
CDhPG enables favourable interphase reconstruction and corrosion suppression at –40 °C. a) Schematic of Zn anode treatment protocol in 8 m ZnCl_2_ with and without CDhPG under subzero conditions. b, c) Linear sweep voltammetry and Tafel plots at −40 °C showing reduced overpotential and increased exchange current density with CDhPG. d,g) SEM images of Zn surfaces after soaking in electrolytes without (d) and with (g) CDhPG. e) XPS O1*s* spectra showing coordination signals of C─OH and C─O─C species on CDhPG‐treated Zn. f) Arrhenius plots indicating decreased charge transfer activation energy upon CDhPG incorporation. AFM height maps of h) 8 m ZnCl_2_ and i) 8 m ZnCl_2_ with CDhPG treatment. j) Corresponding line profiles comparing surface roughness between two conditions in h) and i). k,l) SEM images highlighting surface degradation in the absence (k) and interphase formation in the presence (l) of CDhPG. m) C1*s* XPS spectra further confirming interfacial CDhPG presence via characteristic carbon signals.

Accordingly, the corrosion resistance of the Zn electrode in different electrolytes (with and without CDhPG) was evaluated under subzero conditions. We compared the Zn metal surfaces treated in pure 8 M ZnCl_2_ and CDhPG‐containing electrolytes after 7 days of soaking at −40 °C (**Figure** **4**a). The electrode exhibited much higher overpotentials at −40 °C than at under 25 °C in both ZnCl_2_‐CDhPG and pure ZnCl_2_ electrolytes, indicating the increased activation energy for HER at low temperatures (Figure S14). As shown in the linear sweep voltammetry (LSV) curves with a scan rate of 5 mV∙s^−1^ at −40 °C (Figure 4b), the Zn electrode in pure electrolyte was found to have a low overpotential (−0.09 V) at 20 mA∙cm^−2^. This overpotential increased to −0.18 V after adding 20 mg mL^−1^ CDhPG in the electrolyte, confirming that CDhPG can effectively suppress the generation of H_2_ on the Zn anode. As shown in Figure 4c, with increasing CDhPG content in the 8 m ZnCl_2_ electrolyte, the corrosion current density gradually decreases, confirming the enhanced corrosion resistance. Such improvement can be attributed to the hydroxy‐rich CDhPG additives that alter the electrode from direct contact with H_2_O and Cl^−^ by the formation of protection layer on Zn anode interface (Figure S15, Supporting Information). The SEM images (Figure 4d,g) reveal that Zn surfaces in the presence of CDhPG remain smooth and compact, in sharp contrast to the severely roughened counterparts in the additive‐free system. X‐ray photoelectron spectroscopy (XPS) analysis of O1*s* spectra (Figure 4e) further supports the formation of a modified interphase: the CDhPG‐containing system displays new peaks corresponding to C─O─C and C─OH bonds, implying that polyhydroxylated CDhPG molecules are anchored to the Zn surface thus can suppress the by‐product generation and Zn anode corrosion. Post‐immersion XPS analysis (Figure S16, Supporting Information) further reveals that the Zn electrode soaked in ZnCl_2_ electrolyte for 7 days exhibits a distinct metal‐O peak in the O1*s* spectrum, indicating the formation of surface metal oxides. In contrast, no new peaks are observed for the electrode immersed in the ZnCl_2_‐CDhPG electrolyte, confirming that the additive effectively suppresses by‐product formation and Zn anode corrosion.

The low‐temperature reaction kinetics of Zn^2+^ ion in ZnCl_2_‐CDhPG electrolyte were further explored. As shown in Figure 1e and Figure S17 (Supporting Information), the ZnCl_2_‐CDhPG electrolyte exhibited a higher ionic conductivity, with much enhanced ionic transport efficiency (112% increase) at ─40 °C. In accordance with this, the electrochemical impedance spectroscopy (EIS) curves at different temperatures, the activation energy of the Zn electrode in different electrolytes can be calculated by the Arehenius equation. As shown in Figure 4f, the associated reduction in activation energy for interfacial charge transfer‐from 16.89 kJ∙mol^−1^ (ZnCl_2_) to 12.39 kJ∙mol^−1^ (ZnCl_2_ + CDhPG) as shown in Arrhenius plots. In addition, compared to the pure ZnCl_2_ electrolyte, the initial reduction potential of Zn^2+^ in the ZnCl_2_‐CDhPG electrolyte is shifted to a low value (Figure S18, Supporting Information), indicating the enhancement of Zn^2+^ transport kinetics by hydroxy‐rich CDhPG additive further confirms the accelerated ion migration kinetics under cold conditions. Ab initio molecular dynamics (AIMD) simulations are constructed to evaluate the Zn^2+^ de‐solvation behavior at ‐40 °C. As shown in Figure S19 (Supporting Information), Zn^2+^ ion gradually dehydrates from the [Zn(H_2_O)_5_+PG_3_]^2+^ structure. After ≈ 2 picoseconds, it has almost completely separated. In comparison, at the same time, the de‐solvation processes for the [Zn(H_2_O)_6_]^2+^ don't occur at the same time point. The above results imply the hdyroxyl‐rich CDhPG additive can effectively promote the Zn^2+^ de‐solvation ability, thus achieving fast Zn^2+^ diffusion and reaction kinetics.

The Zn nucleation and deposition behavior under low‐temperature conditions was further evaluated. Atomic force microscopy (AFM) topographies and corresponding height profiles (Figure 4h–j) confirm distinct morphological differences: the CDhPG‐treated surface exhibits significantly reduced roughness and more homogeneous coverage compared to the untreated control. Chronoamperometry (CA) measurements at −40 °C (Figure S20, Supporting Information) reveal a continuous 3D diffusion response, indicative of uniform Zn deposition at the Zn‐electrolyte interface. SEM images revealed the surface morphology of Zn anodes after deposition at −40 °C and cumulative capacities of 2, 5, and 15 mAh∙cm^−2^ (Figures S21 and S22, Supporting Information). The Zn electrode in the ZnC_2_‐CDhPG electrolyte presented a smooth, dense surface with minimal signs of dendritic growth (Figure 4l). In contrast, the Zn electrode cycled in pure 8 M ZnCl_2_ exhibited a rough, uneven surface with pronounced anisotropic dendritic protrusions (Figure 4k), highlighting the role of CDhPG in promoting uniform Zn deposition. To further investigate deposition dynamics, in situ optical microscopy was employed to monitor Zn plating in different electrolytes. As shown in Figure S23 (Supporting Information), dendritic growth and surface roughening were clearly evident in the pure ZnCl_2_ system; in contrast, the CDhPG‐containing system maintained a stable morphology. Additionally, C1*s* X‐ray photoelectron spectroscopy (XPS) spectra (Figure 4m) revealed interfacial signatures of C─O─C and C─H/C─C bonds, confirming the formation of a CDhPG‐derived coordination interphase. Collectively, these results demonstrate that CDhPG promotes the formation of a robust, coordination‐tuned interphase that suppresses Zn corrosion, inhibits dendrite formation, and facilitates stable ion transport at subzero temperatures.

### 2.4 Cryogenic Electrochemical Stability Enabled by CDhPG under Subzero Conditions

**Figure 5:**
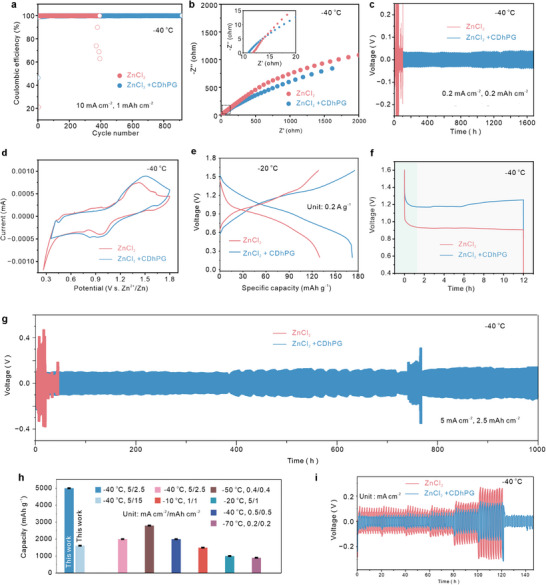
Low‐temperature electrochemical performance of CDhPG‐assisted Zn electrolytes at subzero conditions. a) Coulombic efficiency of Zn||Cu asymmetric cells at 10 mA∙cm^−2^ with/without CDhPG. b) Nyquist plots showing reduced interfacial impedance in CDhPG‐based electrolyte. The inset is the magnification image. c) Voltage profiles of Zn||Zn symmetric cells at 0.2 mA∙cm^−2^ over 1600 h. d) Cyclic voltammetry curves of Zn||V_2_O_5_ full cells showing enhanced redox definition. e) Galvanostatic charge/discharge (GCD) curves of Zn||PANI‐V_2_O_5_ cells with ZnCl_2_ and ZnCl_2_+CDhPG electrolytes at current density of 0.2 A∙g^−1^ and −20 °C (PANI: polyaniline). f) Discharge curves of Zn||V_2_O_5_ at 0.5 A g^−1^. g) Long‐term cycling stability of Zn||Zn symmetric cells at 5 mA∙cm^−2^.over 900 h at −40 °C. h) Discharge capacity comparison with other cryogenic Zn‐based batteries. i) Reversible plating/stripping voltage curves at −40 °C.

To evaluate the practical implications of CDhPG‐assisted electrolyte under extreme subzero conditions, we assessed Zn‐based electrochemical performance at −40 °C. In Zn||Cu asymmetric cells, a high Coulombic efficiency (CE) of ≈99.6% is retained over 850 cycles at 10 mA∙cm^−2^ (1 mAh∙cm^−2^) in CDhPG‐containing electrolyte, whereas the control without CDhPG shows a rapid drop below 80% (**Figure** **5**a). From the EIS plots and the equivalent circuit diagram (Figure 5b; Figure S25, Supporting Information), Zn electrode in ZnCl_2_‐CDhPG electrolyte also shows a smaller charge transfer impedance (Rc) of 44.78 Ω than that in pure ZnCl_2_ electrolyte (9791 Ω), demonstrating that CDhPG additive substantially promotes rapid ion and charge transfer kinetics (Table S3, Supporting Information). In addition, the internal resistance (Rs) of the ZnCl_2_‐CDhPG‐based electrode is as low as 1.601 Ω, compare with the high value of the Zn electrode in pure ZnCl_2_ electrolyte (11.13 Ω). Such low R values indicate the excellent electrical conductivity and remarkable electrochemical activity at low temperature.

Benefiting from the promising dual remodelling effect that simultaneously prohibits Zn anode corrosion and enhances rapid Zn^2+^ reaction kinetics, the electrodes in ZnCl_2_‐CDhPG electrolyte would stimulate superior cycling stability at high rate. As shown in Figure 5c, the symmetric Zn||Zn cells, CDhPG enables highly stable voltage profiles over 1500 h at −40 °C and 0.2 mA∙cm^−2^, in stark contrast to the noisy and short‐lived response of the pristine electrolyte. Cyclic voltammetry (CV, Figure 5d) on Zn||V_2_O_5_ full cells show that the CDhPG electrolyte supports better‐defined redox peaks. In addition, the reaction rate of the ZnCl_2_‐CDhPG‐based ZIB was also relatively high, at a current density range from 0.1 to 5 A∙g^−1^, delivers a specific capacity of 122.6 mAh∙g^−1^ at 0.1 A∙g^−1^, retaining ≈86.4% at 5 A∙g^−1^ (Figure 5e). After being fully charged and rested for 12 h (Figure 5f), ZIB with ZnCl_2_‐CDhPG electrolyte showed a lower voltage drop (0.53 V) than did the pure ZnCl_2_‐based ZIB (0.70 V), indicating improved interfacial stability and ionic mobility at subzero temperatures. Long‐term symmetric cell cycling at −40 °C under high current density (5 mA∙cm^−2^) and deep cycling depth (2.5 mAh∙cm^−2^, 43% DOD) demonstrated excellent durability with a stable voltage profile and negligible degradation over 900 h in the ZnCl_2_‐CDhPG electrolyte (Figure 5g). In contrast, the pristine ZnCl_2_ system exhibits severe voltage fluctuations and early cell failure, highlighting the critical role of CDhPG in stabilizing Zn plating/stripping under extreme conditions. It is worth noting that the transient voltage fluctuations observed in the Zn||Zn symmetric cells (Figure 5c,g) originate from dynamic interfacial relaxation and reversible Zn plating/stripping behavior under cryogenic conditions. At −40 °C, the increased electrolyte viscosity and reduced Zn^2^⁺ diffusivity lead to intermittent ion redistribution and self‐adjustment at the electrode surface. Such minor oscillations reflect a stable interfacial regulation process rather than electrochemical instability, indicating that the CDhPG‐assisted electrolyte enables smooth Zn deposition and stripping with suppressed dendritic growth over prolonged cycling.Post‐cycling XPS analysis (Figure S24, Supporting Information) further validate the formation of a stable coordination‐tuned interphase. The platform also delivers superior discharge capacity (Figure 5h; Table S5, Supporting Information) compared to reported cryogenic Zn‐based batteries, surpassing prior benchmarks^[^ ^34^, ^37^, ^61^ ^]^ even at −40 °C and 5 mA∙cm^−2^. Lastly, reversible voltage curves at −40 °C (Figure 5i) highlight low polarization and smooth Zn plating/stripping, affirming the electrolyte low‐temperature applicability. Collectively, these results demonstrate that CDhPG enables exceptional electrochemical reversibility, rate capability, and long‐term cyclability under harsh cryogenic conditions, by modulating ion solvation, interfacial charge transfer, and dendrite suppression.

## 3 Conclusion

By drawing inspiration from the survival mechanisms of polar‐adapted fish, we developed a bioinspired supramolecular additive (CDhPG) that mimics the function of naturally occurring antifreeze proteins; that is, the CDhPG combines ice‐binding capacity with dehydration behavior similar to that of natural ion channels, thus enabling high‐performance aqueous Zn‐ion batteries at low temperatures. The hydroxyl‐rich branches of CDhPG provide favourable bond distances (≈2.76 Å) for anchoring ice surfaces, while its internal cavities offer a size‐matched coordination environment that facilitates Zn^2+^ desolvation. This dual‐functional configuration allows simultaneous modulation of the ice‐water interface and Zn‐electrolyte interface. Such synergistic interfacial remodelling promotes oriented Zn(002) deposition, suppresses dendrite formation, mitigates corrosion and HER, and accelerates Zn^2+^ transport at subzero temperatures. As a result, the ZnCl_2_‐CDhPG electrolyte enables dendrite‐free cycling over 900 hrs at −40 °C under 5 mA∙cm^−2^ and 43% DOD, with a suppressed freezing point below −40 °C. A full cell incorporating this electrolyte demonstrates stable operation at −40 °C for over 500 cycles with high capacity retention, showcasing its potential for practical energy storage in extreme environments. This molecular strategy may be broadly applicable to other polyhydroxyl compounds, thus offering a new pathway for low‐temperature electrolyte design.

## Conflict of Interest

The authors declare no conflict of interest.

## Supporting information



Supporting Information

Supplemental Video 1

Supplemental Video 2

## Data Availability

The data that support the findings of this study are available from the corresponding author upon reasonable request.
