# Supplementary material for: Hydroxyl‐Rich Hyperbranched Polyglycerol Additive for Low‐Temperature Aqueous Zinc Batteries: Sustained and Efficient Dehydration and High‐Conductivity
Source: Adv Sci (Weinh). 2025 Nov 19;13(7):e16639. doi: 10.1002/advs.202516639 (PMC12866746; doi:10.1002/advs.202516639)
Supplement: Supplementary file 1 — Supporting Information [file ADVS-13-e16639-s001.docx]

Supporting Information

Hydroxyl-Rich Hyperbranched Polyglycerol Additive for Sustainable, Efficient Dehydration and High-Conductivity Low-Temperature Aqueous Zinc Batteries

Xiaoping Li,^a+^ Tan Jin^b+^, Zhiqiang Wang,^c^ Yaoxuan Chen,^a^ Chunlong Yun,^a^ Jie Shang,^a^ Yan Ge,^d^ Tingli Lu,^a*^ Wei Zhuang^b*^, Yue Ma,^c*^ Zhenhui Qi,^e*^

+ Joint First Coauthors

**Table of Contents**

[1.Material S3](#_Toc24725)

[1.1 Synthesis, purification and characterizations of CDhPG S3](#_Toc8474)

[1.2 Prepare of electrolytes S4](#_Toc6509)

[1.3 Characterization S5](#_Toc21500)

[1.4 Electrochemical measurements S6](#_Toc1575)

[1.5 Computational Simulations S7](#_Toc9426)

[2. Physical and Chemical Properties and Solvation Structure S10](#_Toc22845)

[References S40](#_Toc15074)

# 1.Material

## 1.1 Synthesis, purification and characterizations of CDhPG

The complete synthesis pathway is depicted in Scheme S1. A representative synthesis procedure^S1^ is outlined below: Within a glove box, a solution containing β-CD (0.2 g, 0.176 mmol) and 18-crown-6 ether (0.33 g, 1.25 mmol) dissolved in dry DMF (10 mL) was transferred into a 50 mL one-neck flask that already contained sodium hydride (0.0336 g, 1.4 mmol). This mixture was then vigorously stirred for approximately 2 hours at a temperature of 50℃. Subsequently, after elevating the mixture's temperature to 80 ºC, a solution of glycidol (1.6 g, 20.6 mmol) in dry DMF (10 mL) was gradually introduced dropwise into the reaction system over a span of 20 hours. The reaction was halted by adding a small quantity of water, and the resultant reaction mixture underwent dialysis against pure water (with a molecular weight cut-off, MWCO, of 3500 Da) for a duration of one week, followed by vacuum freeze-drying. The final product obtained was a transparent, viscous liquid, with an approximate yield of 50%.

## 1.2 Prepare of electrolytes

The ZnCl_2_-CDhPG electrolytes were formulated by introducing varying concentrations (10 mg and 20 mg) of CDhPG into an 8 M ZnCl_2_ solution. V_2_O_5_ was deposited onto a pristine carbon cloth through electrochemical polymerization. The proportions of the components were set as follows: V_2_O_5_, Conductive Carbon Black SP, and PVDF were mixed at a weight ratio of 70:20:10. Initially, V_2_O_5_ and Conductive Carbon Black were combined, followed by the gradual addition of PVDF dissolved in NMP into the beaker while stirring. After a period of time, the V_2_O_5_ and Conductive Carbon Black mixture was incorporated into the PVDF-NMP solution. Subsequently, the resulting compound was uniformly coated onto the carbon cloth. Electrodeposition was performed using an electrochemical workstation (CHI760E), applying a constant voltage of 0.85 V for a duration of 600 s.

## 1.3 Characterization

The nuclear magnetic resonance (NMR) spectra of 2H was conducted using ADVANCE NEO 500 (Bruker). The Fourier transform infrared spectroscopy (FTIR) spectra was obtained from Tensor II (Bruker, 0.4 cm-1). X-ray photoelectron spectroscopy (XPS) studies were conducted using Kratos (Axis Supra). The microstructures and morphologies of electrodes were characterized by field emission scanning electron microscopy (FE-SEM, Zeiss Gemini SEM 300, 0.02-30.0 kV). Atomic force microscope (AFM) results were obtained from Cypher S (Asylum Research). The contact angle studies were conducted using DSA 25 (KRUSS). The in situ optical observations were conducted using an optical microscope equipped with EMCCD and monochromator.

Analysis of ice recrystallization inhibition (IRI) was conducted by measuring the mean largest grain sizes (MLGS).^S2^ To enable these measurements, a sealed, temperature-controlled cryostage capable of reaching -193 ^o^C while maintaining a nitrogen protective atmosphere was designed. This stage features an observation window on its upper surface to permit light transmission for microscopy. A microscope slide was positioned on the metal block surface and pre-cooled to -100 °C using liquid nitrogen. The cryostage door was then opened, and a 10 μL sample aliquot was deposited using a 20 μL micropipette at the height of 1.5 meter. Upon impacting the pre-chilled glass slide, the droplet instantaneously formed an ice wafer approximately 10 mm in diameter and 10 μm thick, after which the cryostage was rapidly resealed. The cryostage was immediately transferred to a microscope stage and annealed at -6 °C for 30 minutes. Optical images were acquired using a polarized light microscope equipped with a built-in digital camera under a 20× objective lens. The grain areas of the 20 largest ice crystals within the field of view were measured using ImageJ software. For each sample, mean grain area (MGA) was calculated from five images obtained from at least two independent wafers (totaling ≥100 ice crystals). MLGS was assessed by comparing these measurements against the recrystallized ice crystal size obtained from pure water controls.

## 1.4 Electrochemical measurements

All low-temperature tests were carried out in a freezer facility with constant temperature and humidity (YH-80R, -70-150 ℃). 200 μm Zn plate (142.8 mg cm^-2^) was used in most experiments and tests, and 10 μm Zn plate (7.14 mg cm^-2^) was used for the tests at -40 ℃ and full battery. Zn//Cu and Zn//Zn cells were assembled into Swagelok battery mold with ZnCl_2_-CDhPG as the electrolyte to evaluate the coulombic efficiency, cycle performance, nucleation and deposition processes. The current densities were set ranging from 1 to 10 mA cm^-2^ for Zn//Zn cells. The corrosion and hydrogen evolution reaction (HER) performance of Zn electrodes were measured by linear scanning voltammetry at a scan rate of 5 mV s^-1^. For the Zn-ion battery tests, full-cells were assembled using V_2_O_5_ as the cathode, Zn foil as the anode, ZnCl_2_ and ZnCl_2_-CDhPG as the electrolyte, and the voltage range was 0.2-1.8 V under different current densities (0.1-5 A g^-1^). For the large-area pouch cells, V_2_O_5_ was used as the cathode. The cyclic voltammetry (CV) tests, galvanostatic charge/discharge (GCD) curves and electrochemical impedance spectroscopy (EIS, the frequency range from 0.01 Hz to 100 kHz) were conducted on the electrochemical workstation (CHI760E). Rate performance and long cycling tests were measured by the battery test system (Neware). All the controlled samples were tested under the same conditions.

## 1.5 Computational Simulations

The geometric optimizations based on density functional theory (DFT) was carried out using the powerful Gaussian 16 package and the hybrid B3LYP-D3 (BJ) combined with the 6-311G (d,p) basis set was utilized.^S3-5^. The molecular electrostatic potential (ESP) of the β-CD and hyperbranched polyglycerol (hPG) were calculated using the Multiwfn 3.8 program and the visually stunning ESP maps were well presented using the VMD 1.9.4 software.^S6^ The interaction energy was calculated at the M06/def2-TZVP level and the frequency calculations were carried out to check that all the geometries correspond to energy minima. The binding energy $\Delta E_{bind}$ was evaluated using the following equation:

$$\Delta E_{bind}=E\left( complex \right)-[E\left( molecule \right)+E\left( ion/water \right)]$$

Classic molecular dynamics simulations were carried out by the GROMACS 2024.3 to give an atom-level insight of the Zn^2+^ solvation structures in ZnCl_2_-CDhPG and ZnCl_2_-β-CD.^S7^ The CHARMM36 force field plus CHARMM general force field (CgenFF) and the TIP4P/ice water model were used in the simulation.^S8-10^ The initial configurations of electrolyte systems were constructed that the CDhPG or β-CD molecule was centered in a (5 × 5 × 5 nm^3^) cubic simulation box and the 1 M (75 ZnCl_2_), 4 M (300 ZnCl_2_), or 8 M (600 ZnCl_2_) Cl^-^, Zn^2+^, H_2_O were randomly inserted. The integration time step was 2.0 fs during the MD simulations. The LINCS algorithm was used to constrain all bonds to hydrogen atoms, the cutoff radius for calculating Coulomb and van der Waals interactions was 1.0 nm. The long-range electrostatic interactions were calculated using Particle Mesh Ewald (PME) summation. After energy optimization with the steepest descent method (steep) for 50,000 steps, a 10 ns NPT simulation was performed for the equilibrium. Then a 100 ns production simulation was obtained and the configurations were saved every 10ps at 300 K and 1 bar. To avoid quenching effects, we performed a 1.0 K/ns cooling ramp from 300 K to 233 K and thereafter 20 ns NPT simulation was performed for the equilibrium at 233 K. Then a 200 ns production simulation was obtained, in which the temperature was maintained at 233K and the pressure at 1 bar.

Nonequilibrium NVT simulations are perfomed with cells containing slabs of vapor, liquid and ice.^S11^ The simulation cells are consist of one CDhPG molecule and about 4500 water molecules. The size of the simulation box for the system is set to 5.50 × 5.17 × 10.0 nm^3^, in which the primary prismatic plane is exposed to liquid water. Two layers of ice Ih (784 molecules) are generated with the program GenIce2 at the density 0.909 g/cm^3^ of ice Ih for TIP4P/Ice.^S12^ The center of mass of CDhPG is initially placed approximately 2.0 nm above the ice surfaces.The oxygen atoms of the molecules in these two layers of ice are harmonically restrained at their original positions with a force constant of 1000 kJ mol^−1^ nm^−2^. An energy minimization is followed by a 1 ns NVT-MD simulation at 275 K for equilibration. The production NVT MD run is then evolved for 500 ns at 267 K and 233 K. The molecules are identified as ice or liquid using the CHILL+ algorithm.^S13^

Geometric structure optimization based on density functional theory (DFT) was performed using the Vienna Ab initio Simulation Package (VASP).^S14^ The Perdew–Burke–Ernzerhof (PBE) generalized gradient approximation (GGA) was employed to describe the exchange–correlation interactions. To account for van der Waals interactions, Grimme's DFT-D3 dispersion correction method was employed. The Brillouin zone was sampled at the gamma point only. A plane-wave kinetic energy cutoff was 400 eV and the electronic energy convergence criterion was accepted with 10^-5^ eV. The ionic relaxation step regarded as converged after the maximum force on each atom was less than 0.01 eV/Å. The model of periodic DFT calculation consists of a 7×7×4 Zn (002) slab, in which the bottom two layers were fixed while the top two layer were free. One hPG molecule or one water molecule was placed on the Zn surface and a vacuum layer of 15 Å was added along the z-direction to minimize periodic interactions. Differential charge density for structural visualization were shown with VESTA.

Ab initio molecular dynamics (AIMD) simulations were performed using the Vienna Ab initio Simulation Package (VASP). The NVT simulations were applied with a time step of 1 fs and a temperature of 233 K. In a two-layer zinc-metal structure, the bottom one layer was fixed while the top one layer was free, and the optimized structure containing one hPG molecule, one Zn^2+^ ion and five water molecules was placed on the zinc surface. Meanwhile, for comparison, the optimized structure with one Zn^2+^ ion and six water molecules was also putted on the zinc surface. Grimme's DFT-D3 dispersion correction method was used. The plane-wave kinetic energy cutoff was set as 400 eV and the Brillouin zone was sampled at the gamma point only. The electronic energy convergence criterion was considered to 10^-5^ eV and the maximum force convergence criterion on each atom was 0.01 eV/Å.

# Physical and Chemical Properties and Solvation Structure


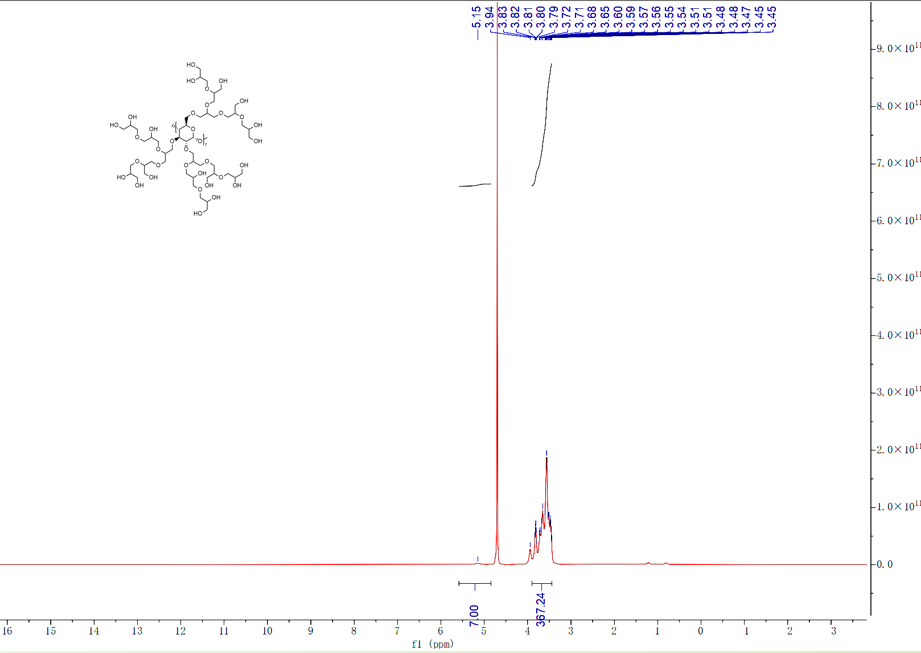


Figure S1a. ^1^H NMR spectrum (400 MHz, D_2_O, 298K) of CDhPG.


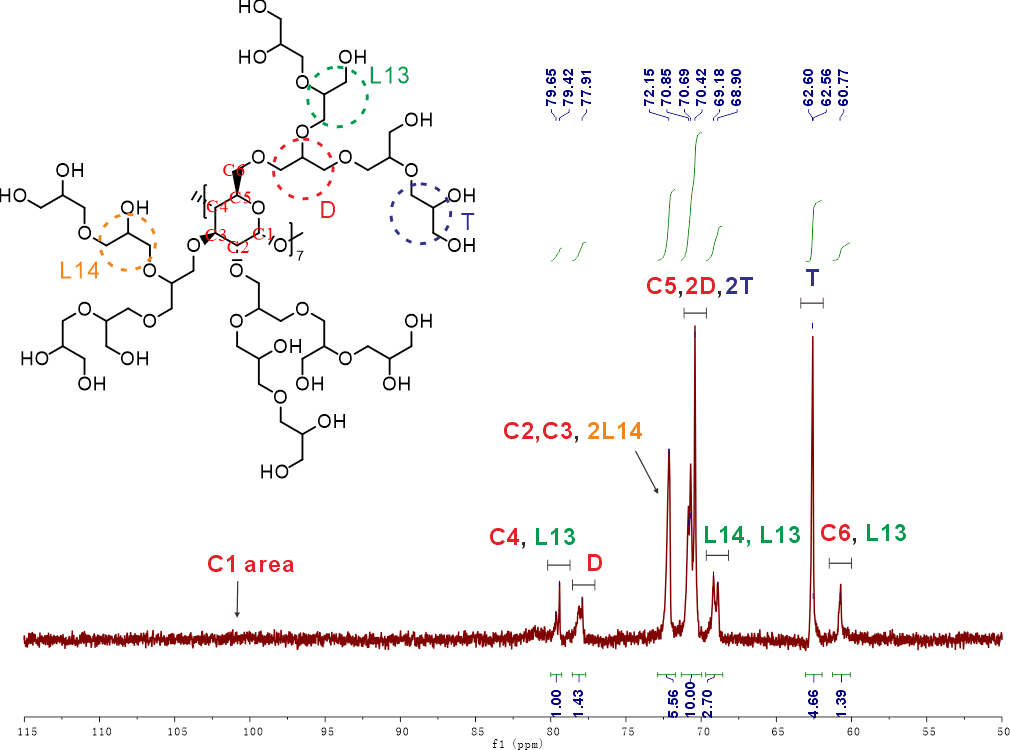


Figure S1b. ^13^C NMR spectrum (400 MHz, D_2_O, 298K) of CDhPG.The signals for C2, C3, L13, L14, and D units can be clearly identified.^S1^


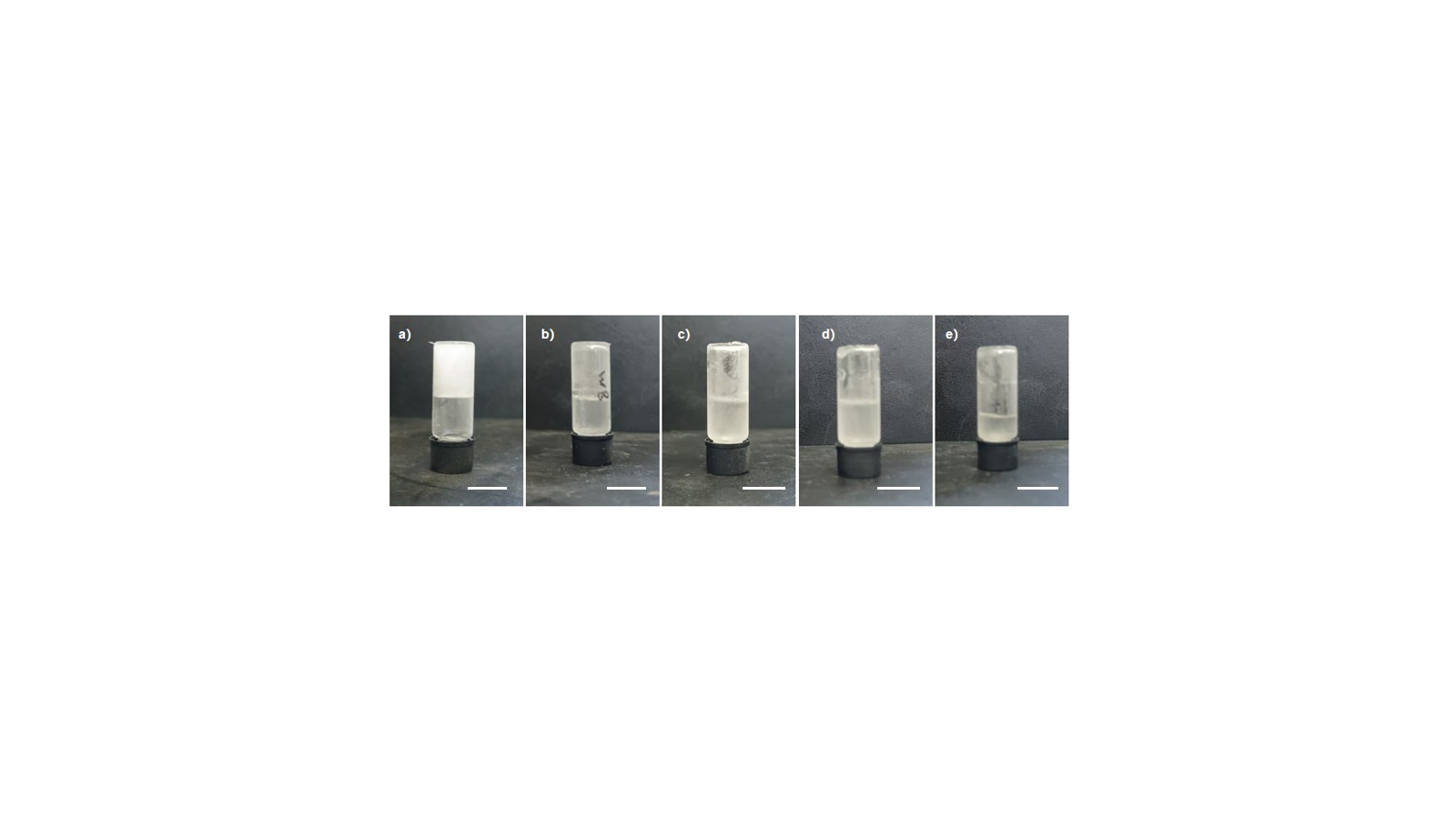


Figure S2. The optical images of ZnCl_2_ electrolytes about a) 4 M ZnCl_2_; b) 8 M ZnCl_2_ c) 8 M ZnCl_2_ + 20 mg/mL CDhPG; d) 12 M ZnCl_2_; e) 12 M ZnCl_2_ + 20 mg/mL CDhPG with different concentrations of hydroxy-rich CDhPG additives at -40 ℃. The Scale bar is 0.5 cm.


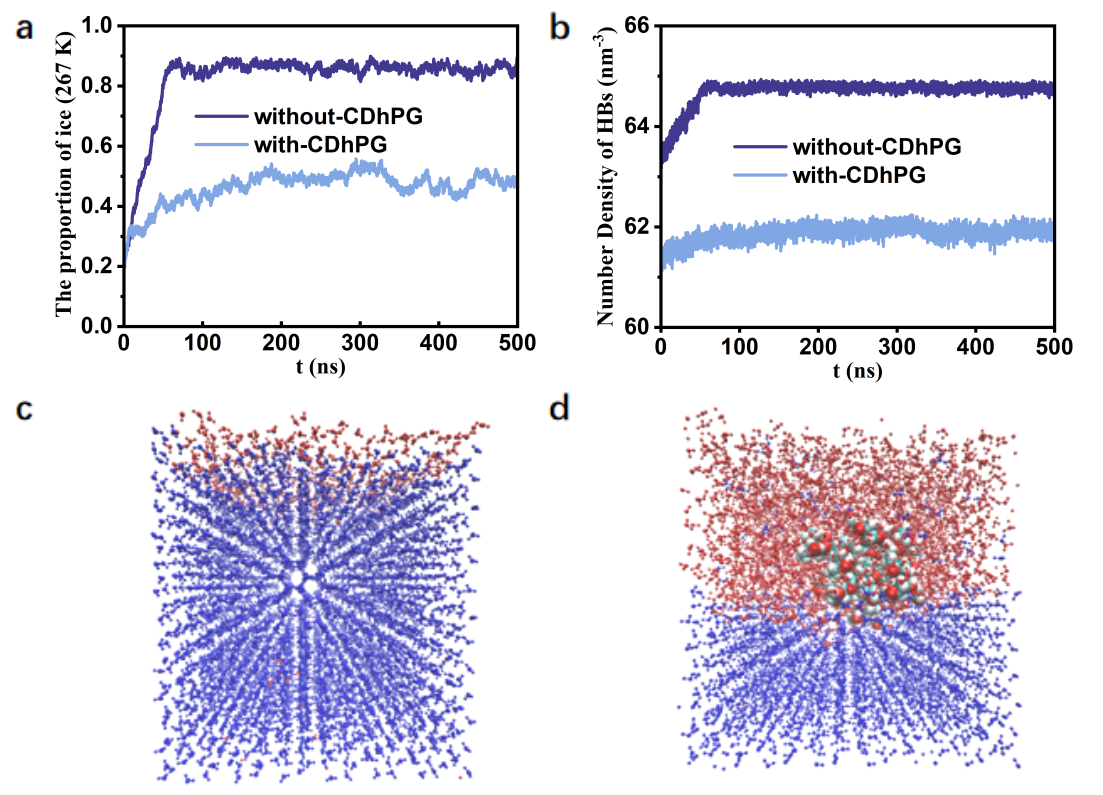


Figure S3 (a) The transformation of liquid water molecules into ice (percentage of ice molecules) and (b) hydrogen bond density over time without/with CDhPG at 267 K. (c-d) Snapshots from molecular dynamics simulations of the ice slab surface without/with CDhPG (presented in VDW style). The ice molecules are colored by bule, the liquid water molecules are showed with red.


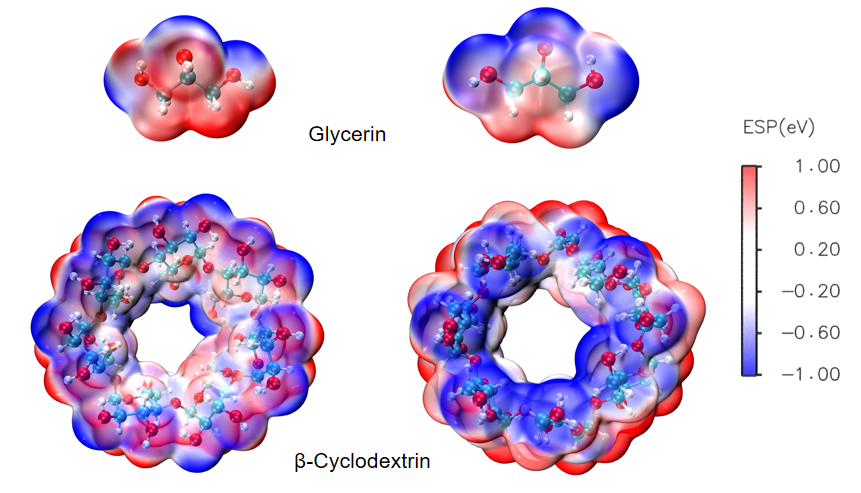


Figure S4. Electrostatic potential (ESP) maps of glycerin and β-cyclodextrin (β-CD) molecules. The ESP distribution is mapped onto the van der Waals surface, with red regions indicating higher positive potential (electron-deficient, electrophilic regions) and blue regions indicating higher negative potential (electron-rich, nucleophilic regions). The potential scale ranges from −1.00 to 1.00 eV.


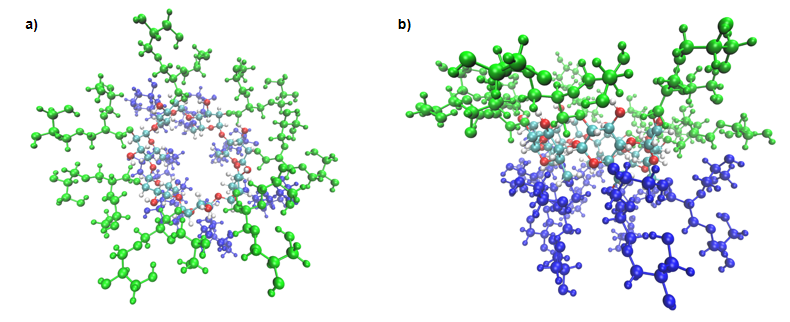


Figure S5. Energy-minimized molecular model of CDhPG obtained from molecular dynamics simulations. The β-CD core is functionalized with hyperbranched polyglycerol (hPG) chains grafted onto the upper rim (green) and bottom rim (blue). (a) Top view. (b) Side view.


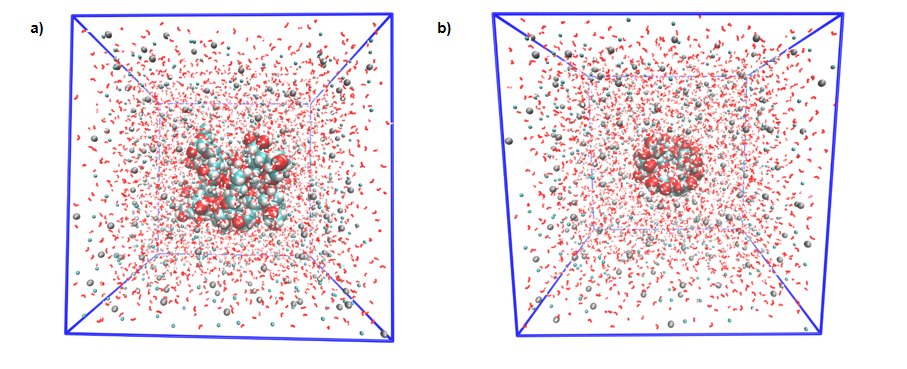


Figure S6. Snapshots from molecular dynamics (MD) simulations of (a) CDhPG and (b) β-CD in ZnCl₂ aqueous solutions with 1M.

Higher ZnCl₂ concentrations of 4 M (300 ZnCl₂) and 8 M (600 ZnCl₂) were also tested. However, at high concentrations, particularly 8 M as used in experiments, the mobility of Zn²⁺ and Cl⁻ ions is markedly reduced due to extensive ion–ion association and the formation of ion clusters, resulting in a highly viscous electrolyte environment. To ensure adequate ion diffusion within the MD timescale, the results presented here correspond to a ZnCl₂ concentration of 1 M.


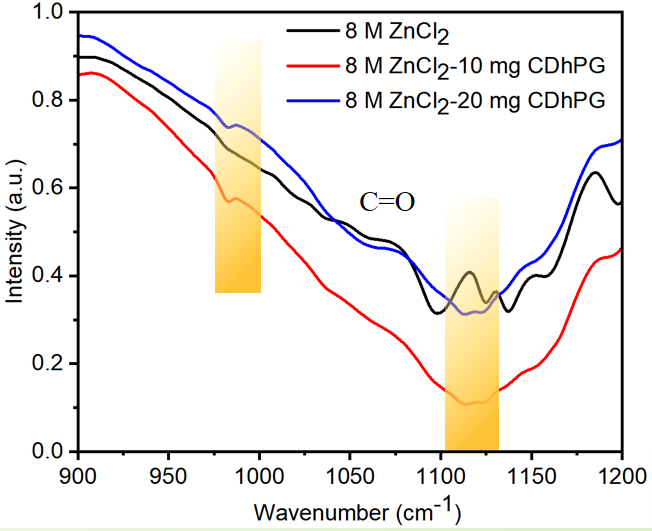


Figure S7. The Fourier transform infrared spectroscopy (FTIR) spectra of C=O stretching for different electrolytes.


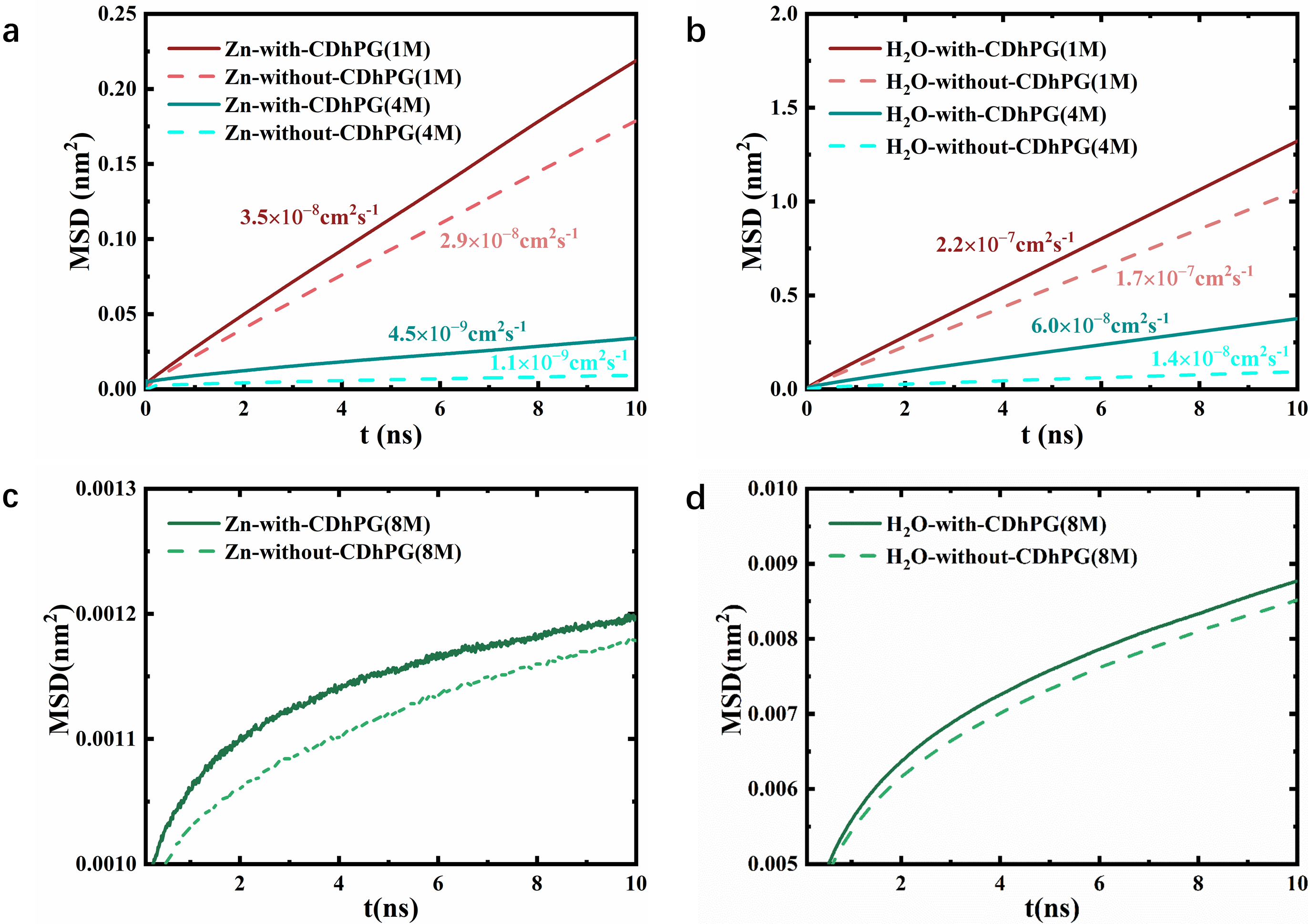


Figure S8. The results of mean square displacement (MSD) of Zn ions (a,c) and water molecules (b,d) with or without CDhPG at 233K for different concentrations of ZnCl_2_.


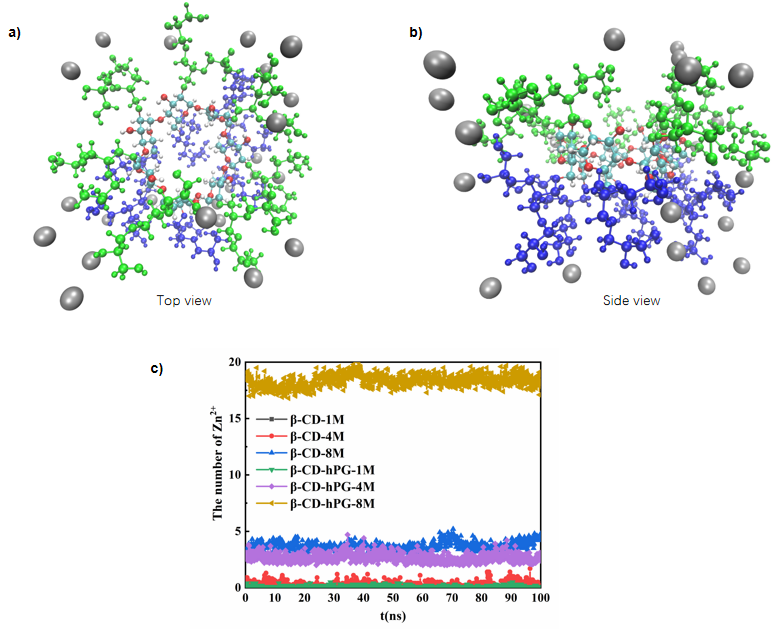


Figure S9. Zn²⁺ adsorption behavior of CDhPG and β-CD in ZnCl₂ aqueous solutions. (a,b) Representative MD snapshots showing Zn²⁺ adsorption from the top view and side view, respectively. Zn²⁺ ions (gray spheres) were defined as being adsorbed when within 3.5 Å of any atom of β-CD or CDhPG. (c) Time evolution of the number of adsorbed Zn²⁺ ions for β-CD and CDhPG at 1 M, 4 M, and 8 M ZnCl₂ concentrations.

Table S1. Statistical analysis of Zn^2+^ adsorption for β-CD and CDhPG at different ZnCl_2_ concentrations from MD simulations. Zn^2+^ ions are considered adsorbed when the distance between any Zn^2+^ ion and any atom of β-CD or CDhPG is < 3.5 Å.

| **System** | **ZnCl₂ Concentration** | **Total Zn²⁺ Count in System** | **Total Adsorbed Zn²⁺ Count** | **Max Adsorbed Zn²⁺ per Frame** |
| --- | --- | --- | --- | --- |
| β-CD | 1 M | 75 × 10,000 | 158 | 2 |
| β-CD | 4 M | 300 × 10,000 | 2,573 | 3 |
| β-CD | 8 M | 600 × 10,000 | 35,794 | 7 |
| CDhPG | 1 M | 75 × 10,000 | 578 | 2 |
| CDhPG | 4 M | 300 × 10,000 | 26,732 | 8 |
| CDhPG | 8 M | 600 × 10,000 | 183,382 | 23 |

The inset table summarizes adsorption statistics, revealing that CDhPG consistently adsorbs far more Zn²⁺ ions than β-CD at all concentrations, due to the abundant hydroxyl-rich binding sites on its hyperbranched polyglycerol chains. This effect is particularly pronounced at high ZnCl₂ concentrations (8 M), where CDhPG maintains strong ion-binding capacity despite the reduced ionic mobility caused by extensive ion–ion association and cluster formation in the highly concentrated electrolyte.

Based on the results in Figure S9 and Table S1, it is evident that across all tested ZnCl_2_ concentrations (1 M, 4 M, and 8 M), CDhPG consistently adsorbs significantly more Zn^2+^ ions than β-CD. This demonstrates that grafting hyperbranched polyglycerol (hPG) introduces abundant coordination sites-primarily hydroxyl groups-that markedly enhance Zn^2+^ binding capacity. As the ZnCl_2_ concentration increases, both the total number of adsorbed Zn^2+^ ions and the maximum number per frame rise substantially. Notably, at 8 M ZnCl_2_, CDhPG reaches a total adsorption count of 183,382 Zn^2+^ ions, with a maximum of 23 ions in a single frame.

At low concentration (1 M ZnCl_2_), β-CD adsorbs a total of 158 Zn^2+^ ions, whereas CDhPG captures 578 ions-approximately 3.7 times higher-indicating that hPG modification significantly enhances Zn^2+^ capture efficiency even under dilute ionic conditions.

At high concentration (8 M ZnCl_2_), CDhPG exhibits a total adsorption count roughly five times greater than that of β-CD, confirming that its multidentate coordination effect remains robust even in high ionic strength environments. Such high adsorption capacity suggests that CDhPG can effectively facilitate Zn^2+^ dehydration in concentrated ZnCl_2_ electrolytes, thereby improving interfacial reaction kinetics.

The physicochemical origin of this behavior lies in the hPG chains, which provide a highly branched three-dimensional architecture densely populated with hydroxyl groups, enabling stable Zn^2+^ binding through multidentate coordination. In concentrated ZnCl_2_ solutions-where strong ion-ion interactions typically hinder Zn^2+^ capture by unmodified β-CD. The flexible structure and high density of coordination sites in hPG mitigate this limitation, preserving efficient ion adsorption.


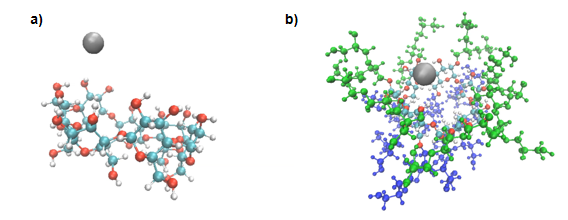


Figure S10. Assessment of Zn^2+^ ion penetration into the β-CD cavity. The penetration radius (rₘₐₓ) was defined as half the maximum distance between the seven primary oxygen atoms of β-CD (blue spheres in the inset). A Zn^2+^ ion was classified as “suspected penetration” if the mass-center distance (r₍cₘ₎) between the ion and the seven oxygen atoms was less than rₘₐₓ. Across all ZnCl₂ concentrations (1 M, 4 M, and 8 M), no Zn^2+^ ions were observed to fully traverse the β-CD channel, as confirmed by the penetration statistics (Table S2). Representative snapshots for (a) β-CD and (b) CDhPG show Zn^2+^ ions (gray spheres) positioned outside the β-CD cavity, verifying that ion transport via direct channel penetration does not occur. Instead, Zn^2+^ binding is dominated by surface coordination to hydroxyl-rich regions rather than trans-cavity passage. See detailed processes in Video S1 and S2.

Table S2. Quantitative analysis of Zn^2+^ ion penetration into β-CD and CDhPG cavities at varying ZnCl_2_ concentrations. **Suspected penetration** refers to Zn^2+^ ions whose mass-center distance (r(c_m_)) from the seven primary oxygen atoms of β-CD is less than the calculated penetration radius (r_max_). **Actual penetration** indicates ions that completely traverse the β-CD channel. Across all tested conditions, no Zn^2+^ ions exhibited complete channel penetration, confirming that direct ion transport through the β-CD cavity is not a viable pathway.

| **Host molecule** | **ZnCl₂ concentration** | **Total Zn²⁺ counts (frames)** | **Suspected penetration counts** | **Actual penetration counts** |
| --- | --- | --- | --- | --- |
| β-CD | 1 M | 75 × 10,000 | 420 | 0 |
| β-CD | 4 M | 300 × 10,000 | 2,600 | 0 |
| β-CD | 8 M | 600 × 10,000 | 555 | 0 |
| β-CD-hPG | 1 M | 75 × 10,000 | 58 | 0 |
| β-CD-hPG | 4 M | 300 × 10,000 | 4,694 | 0 |
| β-CD-hPG | 8 M | 600 × 10,000 | 2,963 | 0 |

Figure S11. Adsorption energy of the Zn-PG_3_ and Zn-H_2_O on Zn surface. PG_3_: a trimer form of polyglycerol.


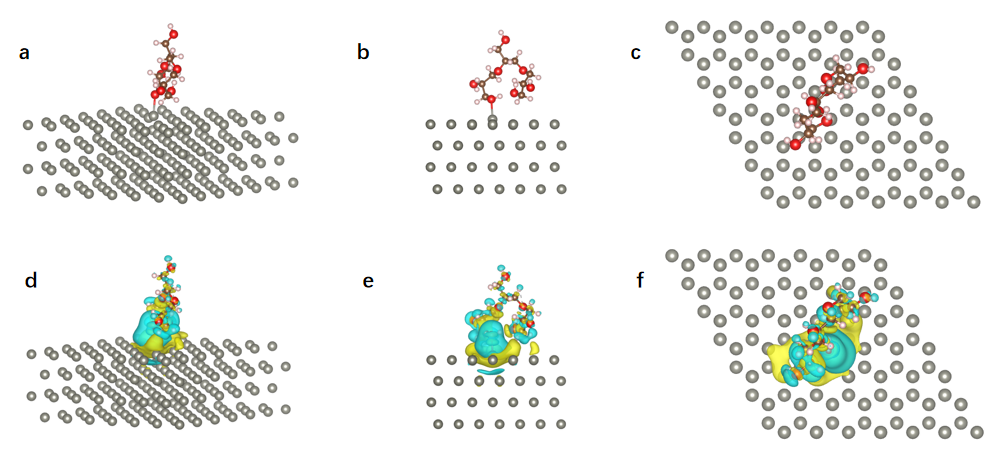


Figure S12. (a-c) Adsorption models and (d-f) charge density difference of Zn slab with the PG_3_ molecule (core functional group in CDhPG) at different viewing angles. The cyan indicates the gain of electrons, while yellow indicates the loss of electrons.


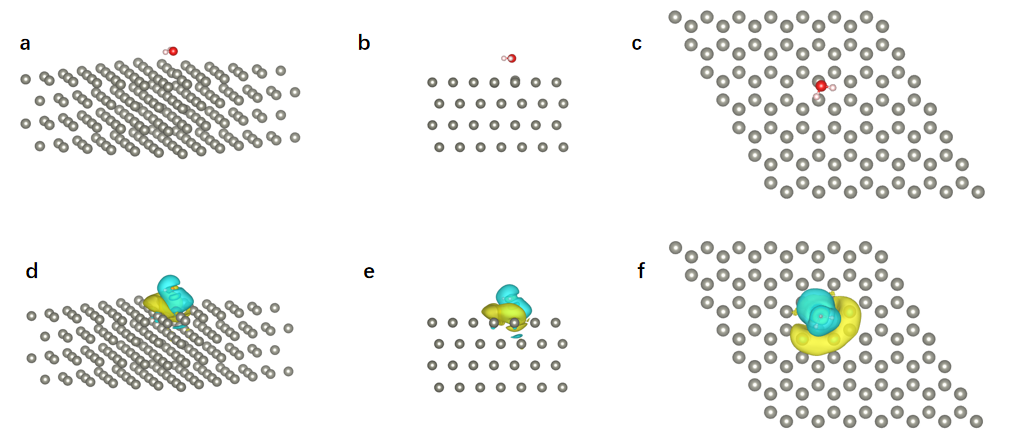


Figure S13 (a-c) Adsorption models and (d-f) charge density difference of Zn slab with the water molecule at different viewing angles. The cyan indicates the gain of electrons, while yellow indicates the loss of electrons.


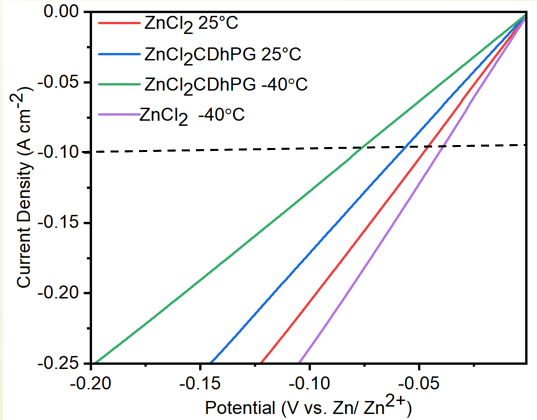


Figure S14. The HER curves of Zn electrodes in different electrolytes at 25 and -40℃.


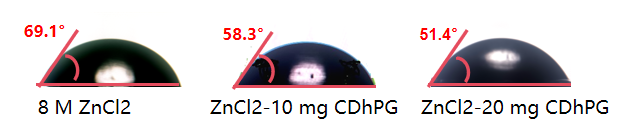


Figure S15.Contact angles of ZnCl_2_ electrolyte on initial Zn plate. Contact angles of ZnCl_2_ electrolyte on Zn electrode after soaking in b) pure ZnCl_2_, c) ZnCl_2_-20 mg/mL CDhPG electrolytes for 7 days at -40 °C.

The contact angle of the Zn electrode after soaking in the ZnCl_2_-CDhPG electrolyte become lower than that of the initial Zn plate, indicating adsorbed shield layer of hydroxyl-rich CDhPG on Zn surface.


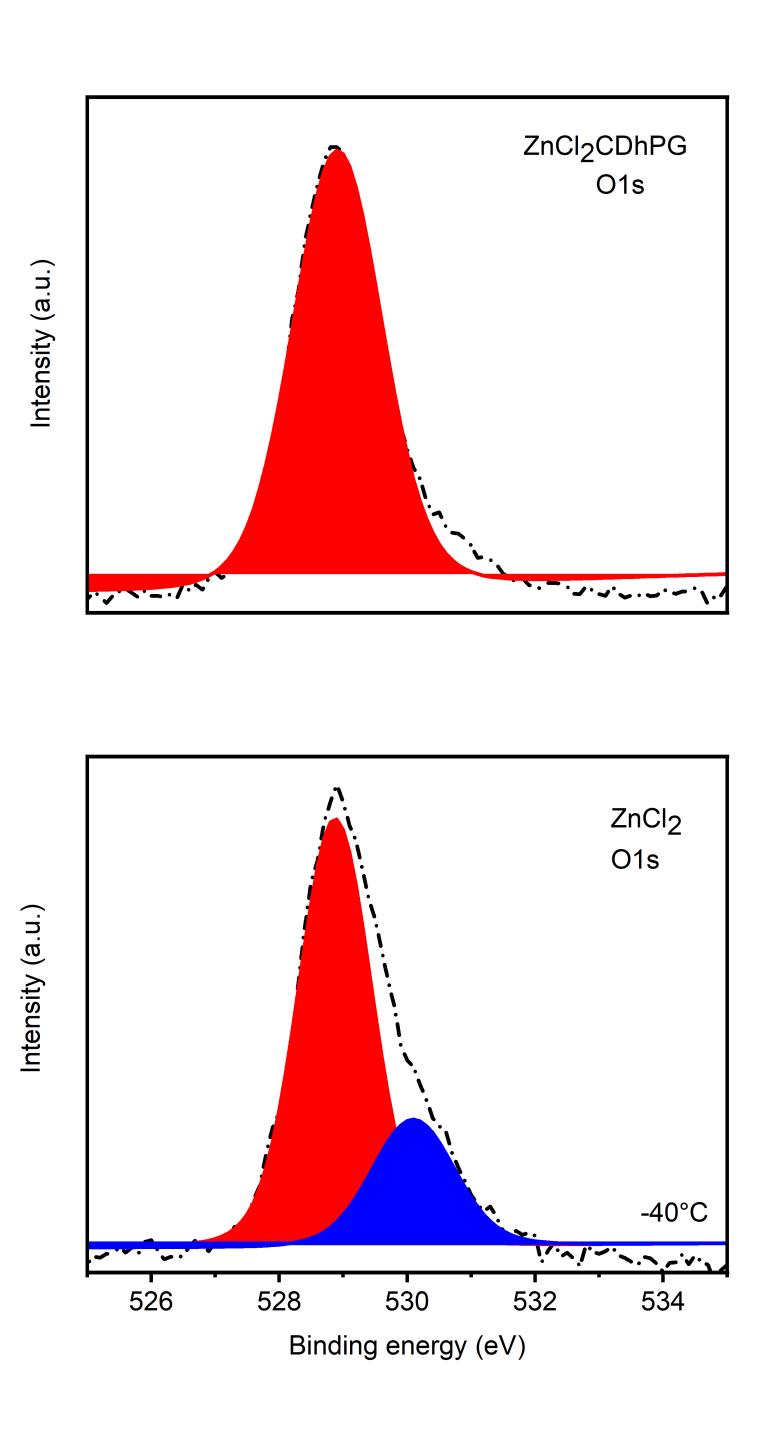


Figure S16. O1*s* XPS spectra of Zn electrodes after immersion at −40 ^o^C in (top) ZnCl_2_ + CDhPG and (bottom) ZnCl₂ electrolytes.

The ZnCl_2_ + CDhPG sample exhibits a dominant Zn–O component with suppressed high-binding-energy –OH species, indicating the formation of a uniform coordination interphase that mitigates surface oxidation and enhances interfacial stability.


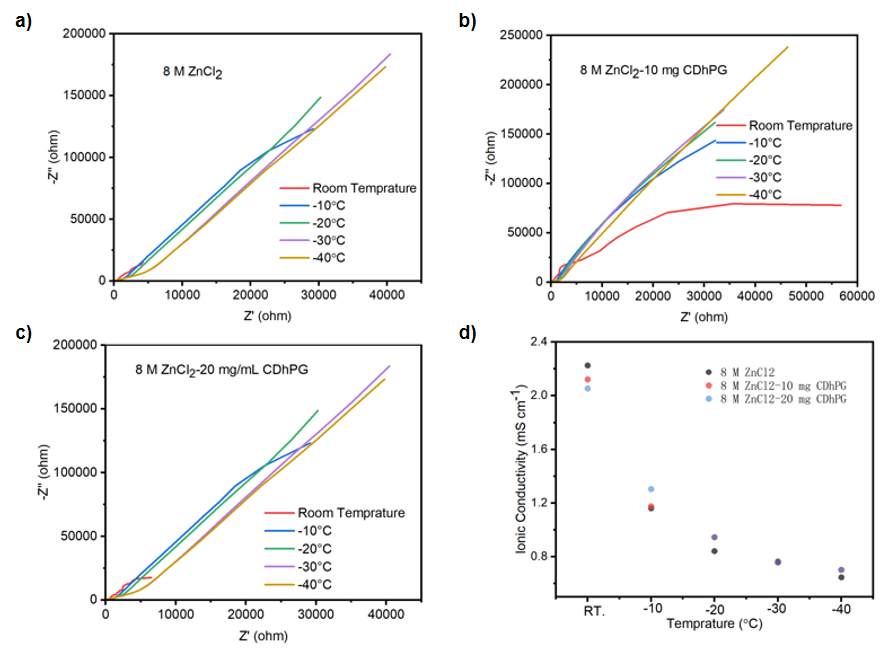


Figure S17. The electrochemical impedance spectroscopy (EIS) results of the a) ZnCl_2_-CDhPG and b) pure ZnCl_2_ electrolytes at different temperatures. c) The ionic conductivity of the ZnCl_2_-CDhPG and pure ZnCl_2_ electrolytes at different temperatures. d) The activation energy of different electrolytes.


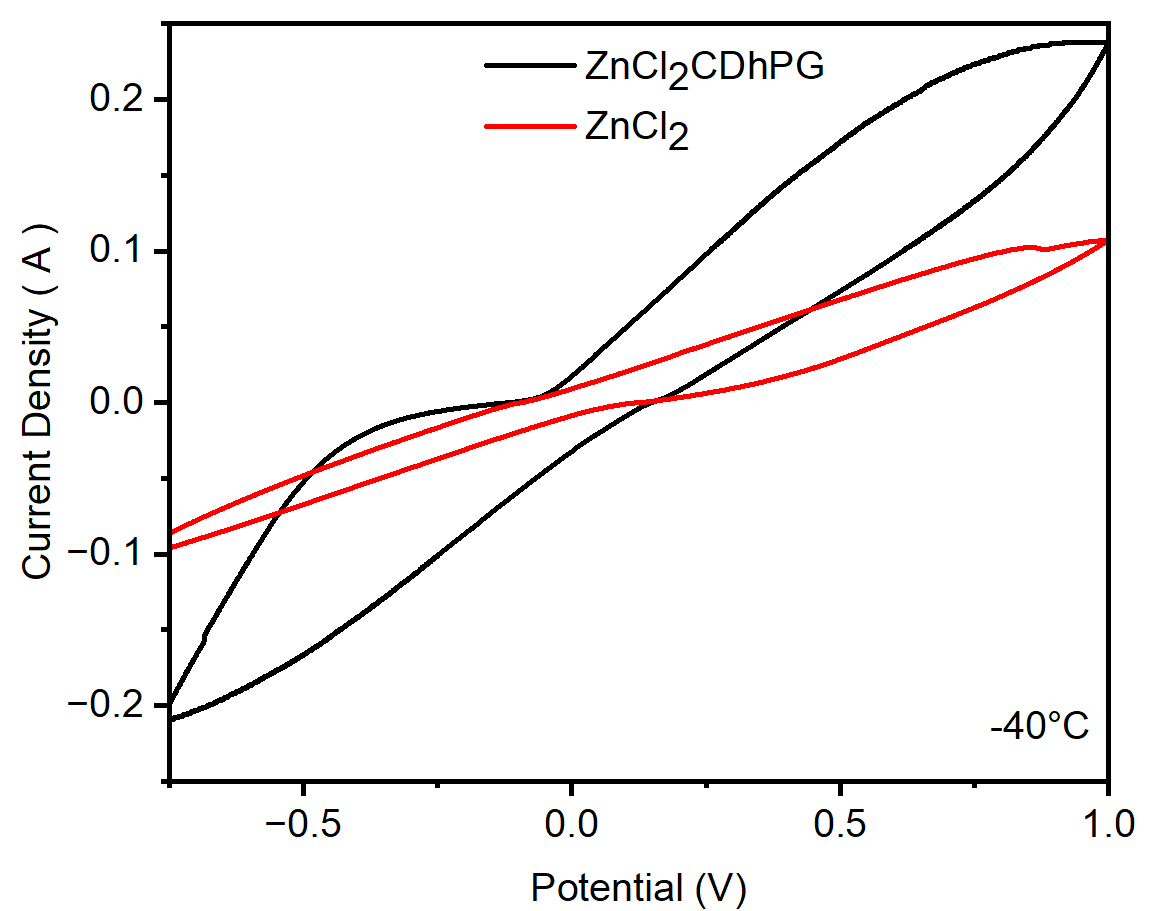


Figure S18. Cyclic voltammetry (CV) curves for Zn//Cu cells in ZnCl_2_-CDhPG and pure ZnCl_2_ electrolytes at -40℃.


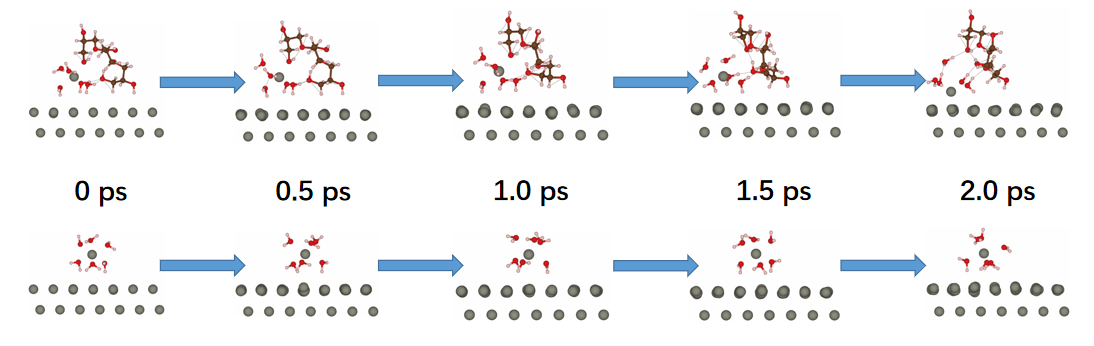


Figure S19. Ab initio molecular dynamics (AIMD) simulations of the de-solvation processes for [Zn(H_2_O)_5_+PG_3_]^2+^ and [Zn(H_2_O)_6_]^2+^ at 233K.


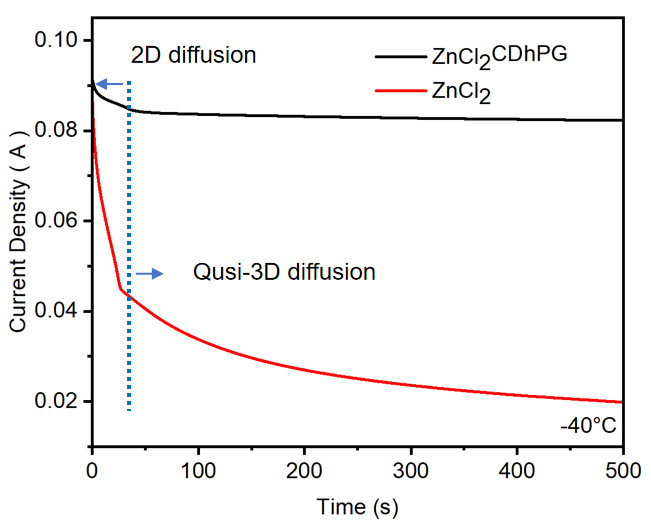


Figure S20. Chronoamperometry (CA) curves of Zn electrodes in ZnCl_2_-CDhPG and pure ZnCl_2_ electrolytes at -40℃.


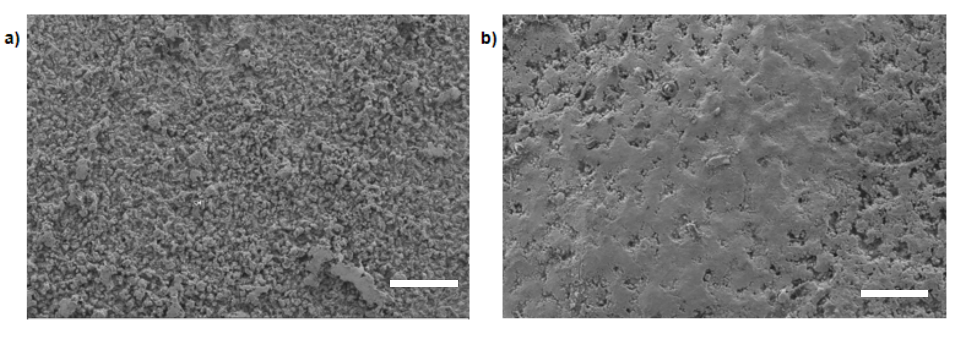


Figure S21. The scanning electron microscopy (SEM) images of Zn electrodes after deposition with a, b) 15 mAh cm^-2^ capacities in pure ZnCl_2_ electrolyte and ZnCl_2_-CDhPG at -40 °C. Scale bar, 100 μm.


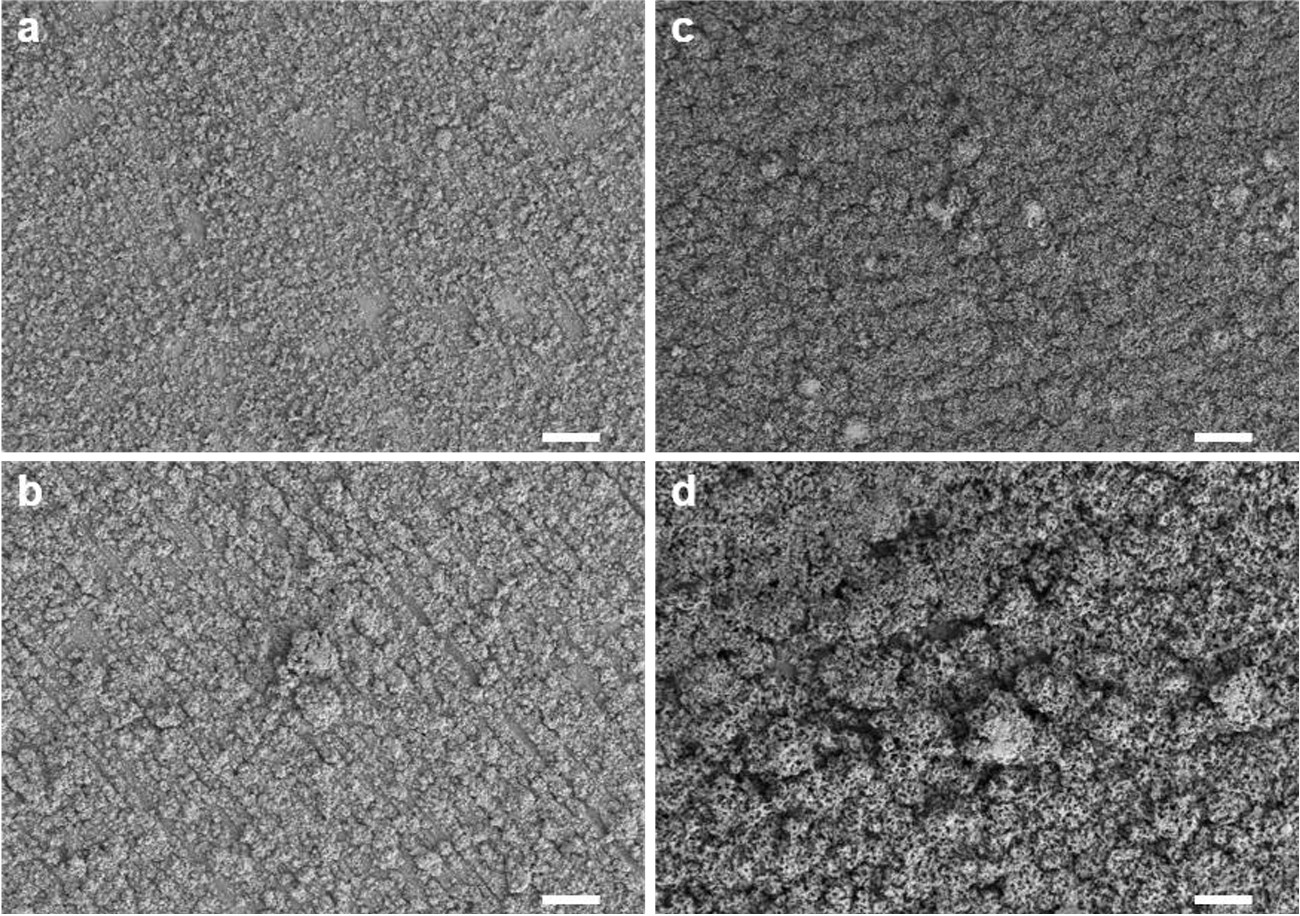


Figure S22. The scanning electron microscopy (SEM) images of Zn electrodes after deposition with a, b) 2 mAh cm^-2^ and c, d) 5 mAh cm^-2^ capacities in pure ZnCl_2_ electrolyte at -40°C. Scale bar, 100 μm for (a, c), 50 μm for (b, d).


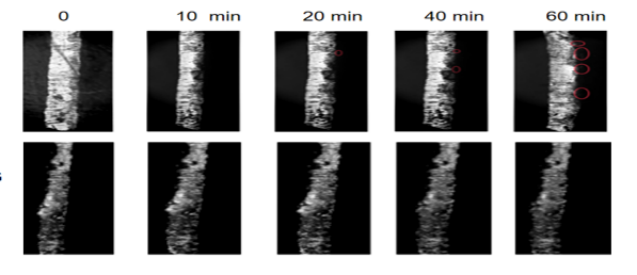


Figure S23. In situ optical observations of Zn electrodes with pure ZnCl_2_ and ZnCl_2_-CDhPG electrolytes in symmetric transparent cells at 5 mAcm^-2^.


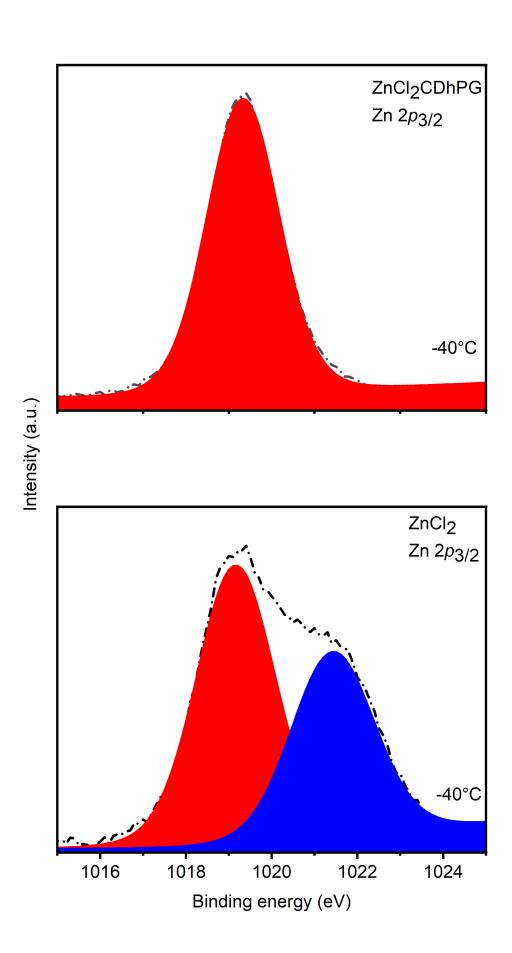


Figure S24. Zn 2*p*_3/2_ XPS spectra of Zn electrodes after long-term cycling at −40 °C. The ZnCl_2_ + CDhPG cell (top, 1000 h at 5 mA cm⁻², 2.5 mAh cm⁻²) shows a single metallic Zn^0^ component, whereas the ZnCl_2_ cell (bottom, failed < 100 h) exhibits an additional high-binding-energy Zn^2+^ peak, indicating severe surface oxidation. These results confirm that CDhPG forms a stable coordination interphase, effectively suppressing side reactions and ensuring long-term cycling stability.


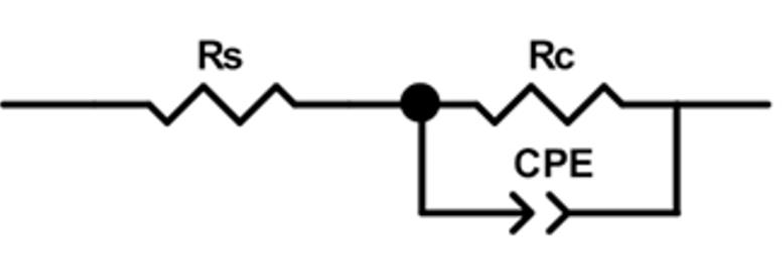


Figure S25. The fitted equivalent circuit diagram of symmetric batteries.

Table S3 Fitting results of the EIS for symmetric cells with different electrolytes

| **Symmetric Cells** | **Rs (Ω)** | **Rc (Ω)** |
| --- | --- | --- |
| 8 M ZnCl₂ +  20 mg/mL CDhPG electrolyte | 1.601 | 9.791 |
| 8M ZnCl₂ electrolyte | 11.13 | 44.78 |

Rs: Solution resistance; Rc: Charge-transfer resistance

Table S4 Long-term galvanostatic cycling of Zn||Zn symmetric cells with ZnCl_2_ and ZnCl_2_ + CDhPG electrolytes at −40 °C under different conditions: 5 mA cm^-2^, 2.5 mAh cm^-2^ and 0.2 mA cm^-2^, 0.2 mAh cm^-2^. The table lists the fitted EIS parameters, showing that CDhPG dramatically reduces both the solution resistance (Rs = 2.59 Ω) and charge-transfer resistance (Rp = 0.97 Ω), confirming improved interfacial conductivity and cryogenic electrochemical stability.

| **Symmetric Cells**  **(mA cm^-2^, mAh cm^-2^)** | **Rs**  **(Ω)** | **CPE-T**  **(Ω^-1^•s^p^)** | **CPE-P** | **Rp**  **(Ω)** |
| --- | --- | --- | --- | --- |
| 5, 2.5 | 2.59 | 0.037 | 0.664 | 0.97 |
| 0.2, 0.2 | 9.58 | 0.0079 | 0.312 | 39.33 |

Rs:Solution resistance; CPE-T: Fitted capacitance parameter of the constant phase element (CPE); CPE-P: Phase exponent of the constant phase element; Rp: Charge-transfer resistance

Table S5. Comparison of low-temperature cycling performance for various ZnCl₂-based electrolytes.

| **Electrolyte system** | **Current density / (mA·cm^-2^)** | **Cycling capacity / (mAh·cm^-2^)** | **Cumulative capacity / (mAh·cm^-2^)** | **Temperature / °C** | **Ref.** |
| --- | --- | --- | --- | --- | --- |
| ZnCl_2_–CDhPG (this work) | 5 | 2.5 | 2500 | –40 | This work |
| ZnCl_2_–CDhPG (this work) | 5 | 15 | — | –40 | This work |
| ZnCl_2_–αDG | 5 | 1 | 5000 | –25 | S[2] |
| ZnCl_2_–in* (MeOH/CH_2_Cl_2_) | 1 | 1 | 1500 | –30 | S15[3] |
| 7.5 M ZnCl_2_ | 0.2 | 0.2 | 45 | –70 | S16[4] |
| *ZnCl_2_–in: literature-reported ZnCl₂-containing electrolyte in a MeOH/CH_2_Cl_2_ mixed solvent. | | | | | |

# References

S1. W. Tao, Y. Liu, B. Jiang, S. Yu, W. Huang, Y. Zhou, D. Yan, J. Am. Chem. Soc. **2012**, 134, 762–764.

S2. F. Bu, Y. Gao, W. Zhao, Q. Cao, Y. Deng, J. Chen, J. Pu, J. Yang, Y. Wang, N. Yang, T. Meng, X. Liu, C. Guan, *Angew. Chem. Int. Ed.* **2024**, *63*, e202318496.

S3 Gaussian 16 Rev. C.01; Wallingford, CT, 2016. (accessed).

S4 J. Witte, M. Goldey, J. B. Neaton, M. Head-Gordon, *J. Chem. Theory Comput.* **2015**, *11*, 1481-1492..

S5 L. Goerigk, A. Hansen, C. Bauer, S. Ehrlich, A. Najibi, S. Grimme, *Phys. Chem. Chem. Phys.* **2017**, *19*, 32184-32215..

S6 T. Lu, *J. Chem. Phys.* **2024**, *161*, 082503.

S7 M. J. Abraham, T. Murtola, R. Schulz, S. Páll, J. C. Smith, B. Hess, E. Lindahl, *SoftwareX* **2015**, *1-2*, 19-25.

S8 R. B. Best, X. Zhu, J. Shim, P. E. Lopes, J. Mittal, M. Feig, A. D. Mackerell, Jr., *J. Chem. Theory Comput.* **2012**, *8*, 3257-3273.

S9 J. Huang, S. Rauscher, G. Nawrocki, T. Ran, M. Feig, B. L. de Groot, H. Grubmuller, A. D. MacKerell, Jr., *Nat. Methods* **2017**, *14*, 71-73.

S10 J. L. Abascal, E. Sanz, R. Garcia Fernandez, C. Vega, *J. Chem. Phys.* **2005**, *122*, 234511.

S11 K. Mochizuki, V. Molinero, *J. Am. Chem. Soc.* **2018**, *140*, 4803-4811.

S12 M. Matsumoto, T. Yagasaki, H. Tanaka, *J. Chem. Inf. Model.* **2021**, *61*, 2542-2546.

S13 A. H. Nguyen, V. Molinero, *J. Phys. Chem. B* **2015**, *119*, 9369-9376.

S14 G. Kresse, J. Furthmuller, *Phys. Rev. B Condens. Matter.* **1996**, *54*, 11169-11186..

S15 S3. H. Du, X. Qi, L. Qie, Y. Huang, *Adv. Funct. Mater.* **2023**, *33*, 2302546.

S16 S4. Q. Zhang, Y. Ma, Y. Lu, L. Li, F. Wan, K. Zhang, J. Chen, *Nat. Commun.* **2020**, *11*, 4463.
